# Effect of Psychiatric Advance Directives Facilitated by Peer Workers on Compulsory Admission Among People With Mental Illness: A Randomized Clinical Trial

**DOI:** 10.1001/jamapsychiatry.2022.1627

**Published:** 2022-06-06

**Authors:** Aurélie Tinland, Sandrine Loubière, Frederic Mougeot, Emmanuelle Jouet, Magali Pontier, Karine Baumstarck, Anderson Loundou, Nicolas Franck, Christophe Lançon, Pascal Auquier

**Affiliations:** 1 CEReSS–Health Service Research and Quality of Life Center (UR 3279), Aix-Marseille University, School of Medicine–La Timone Medical Campus, Marseille, France; 2 Department of Psychiatry, Assistance Publique–Hôpitaux de Marseille, Marseille, France; 3 Support Unit for Clinical Research and Economic Evaluation, Department of Clinical Research and Innovation, Assistance Publique–Hôpitaux de Marseille, Marseille, France; 4 ENSEIS, Centre Max Weber (UMR 5283), Lyon, France; 5 Laboratoire de recherche en Santé Mentale et Sciences Humaines et Sociales (Labo SM-SHS), GHU Paris Psychiatry Neurosciences, Paris, France; 6 Resource Center of Psychosocial Rehabilitation, Centre Hospitalier Le Vinatier, Lyon, France; 7 UMR 5229, Université de Lyon and CNRS, Villeurbanne, France

## Abstract

**Importance:**

Reducing the use of coercion in mental health care is crucial from a human rights and public health perspective. Psychiatric advance directives (PADs) are promising tools that may reduce compulsory admissions. Assessments of PADs have included facilitation by health care agents but not facilitation by peer workers.

**Objective:**

To determine the efficacy of PADs facilitated by peer workers (PW-PAD) in people with mental disorders.

**Design, Setting, and Participants:**

A multicenter randomized clinical trial was conducted in 7 French mental health facilities. Adults with a *DSM-5* diagnosis of schizophrenia, bipolar I disorder, or schizoaffective disorder who had a compulsory admission in the past 12 months and the capacity to consent were enrolled between January 2019 and June 2020 and followed up for 12 months.

**Interventions:**

The PW-PAD group was invited to fill out a PAD form and meet a peer worker who was trained to assist in completing and sharing the form with relatives and psychiatrists.

**Main Outcomes and Measures:**

The primary outcome was the rate of compulsory admission at 12 months after randomization. The overall psychiatric admission rate, therapeutic alliance, quality of life, mental health symptoms, empowerment, and recovery outcomes were also investigated.

**Results:**

Among 394 allocated participants (median age, 39 years; 39.3% female; 45% with schizophrenia, 36% bipolar I disorder, and 19% schizoaffective disorder), 196 were assigned to the PW-PAD group and 198 to the control group. In the PW-PAD group, 27.0% had compulsory admissions compared with 39.9% in the control group (risk difference, −0.13; 95% CI, −0.22 to −0.04; *P* = .007). No significant differences were found in the rate of overall admissions, therapeutic alliance score, and quality of life. Participants in the PW-PAD group exhibited fewer symptoms (effect size, −0.20; 95% CI, −0.40 to 0.00), greater empowerment (effect size, 0.30; 95% CI, 0.10 to 0.50), and a higher recovery score (effect size, 0.44; 95% CI, 0.24 to 0.65), compared with those in the control group.

**Conclusions and Relevance:**

Peer worker–facilitated PADs are effective in decreasing compulsory hospital admissions and increasing some mental health outcomes (self-perceived symptoms, empowerment, and recovery). Involving peer workers in the completion of PADs supports the current shift of mental health care from substitute decision-making to supported decision-making.

**Trial Registration:**

ClinicalTrials.gov Identifier: NCT03630822

## Key Points

**Question:**

Do psychiatric advance directives facilitated by peer workers (PW-PAD) reduce coercion and improve clinical outcomes among people with schizophrenia, bipolar I disorder, or schizoaffective disorder?

**Findings:**

In this randomized clinical trial, 394 adults with schizophrenia, bipolar I disorder, or schizoaffective disorder with a previous compulsory hospitalization were randomized into 2 groups (ratio 1:1). Participants in the PW-PAD group experienced significantly fewer compulsory admissions than those in the control group.

**Meaning:**

These findings support the use of peer worker–facilitated psychiatric advance directives to prevent compulsory rehospitalization in people with severe mental illness.

## Teaser

This randomized clinical trial examines 12 months of follow-up data on rehospitalization and mental health outcomes for patients with schizophrenia, bipolar I disorder, or schizoaffective disorder who completed psychiatric advance directives with help from peer workers.

## Introduction

Respect for patient autonomy is such a strong pillar of health care that involuntary treatment should be unusual. Despite this ethical and clinical principle, compulsory psychiatric admissions are far too common in countries of all income levels.^1,2,3^

In the last 20 years, several randomized clinical trials (RCTs) have assessed the effectiveness of interventions in reducing compulsory psychiatric admissions, and systematic reviews showed that the most effective were psychiatric advance statements.^4^ Psychiatric advance statements are written documents that allow adults who have mental illness to state their will and preferences in advance so their choices can be applied if further mental health crises impair their decision-making capacity. A meta-analysis of the 5 most robust RCTs on this topic was published in 2019.^5^ Molyneaux et al^5^ suggested that (1) psychiatric advance statements reduced the risk of compulsory admission among individuals with mental disorders by 25% compared with usual care; (2) RCTs did not provide firm conclusions on other criteria such as therapeutic alliance or psychiatric outcomes; and (3) similar and higher effectiveness was found in pooled studies that addressed interventions with crisis planning and facilitation by health care professionals.

Other research highlights the importance of facilitation in completing psychiatric advance statements,^6^ but having health care professionals serve as facilitators is not an obvious choice. Indeed, a climate of coercion in psychiatry has been described, with informal coercion and use of power beyond formal coercion, that is, involuntary admissions and compulsory treatment.^7,8^ The relationship between patients and health care professionals is permeated by this coercive climate more than clinicians realize.^9^ Because autonomy and self-determination are the main tenets of psychiatric advance statements,^10^ this system can be improved if facilitation is provided by other types of professionals who are less likely to exert undue influence. To date, RCTs have assessed facilitation by researchers^11^ and patient advocates,^12^ but their results are not as significant as those where health care professionals were the facilitators.

In France, advance directives were created by law in 2005 and primarily used in end-of-life health care. Psychiatric advance directives (PADs) have been used without a formal legal or practical framework. We hypothesized that PADs could be implemented with facilitation by peer workers, ie, people with personal experiences of mental distress and psychiatric services who are employed and trained to support others.^13^ Interest in the facilitation of PADs by peer workers is increasing, and a comparison between peer workers and health care agents found no differences in PAD completion rate and quality.^14^ Recent studies showed that PADs facilitated by peer workers were more prescriptive than those facilitated by nonpeer clinicians and had high feasibility and consistency as rated by experts.^15^

To our knowledge, no study has been conducted of the effect on clinical outcomes of peer worker–facilitated PADs (PW-PADs). The current study addresses this evidence gap to determine whether PW-PADs for people with severe mental illness reduce compulsory admissions and provide significant benefits in terms of therapeutic alliance, quality of life, mental illness symptoms, empowerment, and recovery.

## Methods

### Ethics

The trial was submitted and approved June 6, 2018, by the French ethics committee Sud-Ouest et Outre-mer 4 (2018-A00146-49). The study was conducted in compliance with the Declaration of Helsinki, sixth revision; Good Clinical Practice guidelines; and local regulatory requirements. The participants provided both oral and written consent before their enrollment and allocation to the study groups.

### Trial Design

The study, which has the English name description Peer Worker–Facilitated Psychiatric Advance Directive Study (French acronym DAiP), was a multicenter nonblinded RCT conducted in 7 mental health facilities (aka centers) of 3 cities (aka sites) in France (Lyon, Paris, and Marseille) between January 2019 and June 2021. Participants were referred by their treating psychiatrists in mental health institutions. At the time of study inclusion, most participants were discharged from the hospital, but some were still hospitalized. Psychiatrists checked the eligibility criteria and referred eligible participants to research assistants. Research assistants and psychiatrists reviewed the patient information and validated the inclusion and exclusion criteria. Research assistants met participants from both groups at a location of their choice for face-to-face interviews at the time of inclusion and at 6 and 12 months. The 12-month follow-up timeline started directly after randomization. The recruitment period was planned for 12 months but extended by 6 months because of the COVID-19 pandemic. The study was stopped as originally planned 12 months after the last recruitment. Full details are available in the published protocol (Supplement 2).^16^

The study is registered at ClinicalTrials.gov (NCT03630822). This article follows the Consolidated Standards of Reporting Trials (CONSORT) reporting guidelines.

### Population and Randomization

Eligible participants were older than 18 years; were involuntarily admitted to the hospital within the past 12 months; had a diagnosis of schizophrenia, bipolar I disorder, or schizoaffective disorder according to *DSM-5* criteria^17^; had decision-making capacity assessed by a psychiatrist according to the MacArthur Competence Assessment Tool for Clinical Research^18^; were covered by French government health insurance; and understood French. The exclusion criteria included being considered unable to provide informed consent and being under tutorship (the more restrictive of 2 levels of guardianship in France).

Immediately after signing the consent form, participants were randomly assigned using a web-based system at a 1:1 ratio. The randomization list used a permuted block design and was stratified by the center. Research assistants, treating clinicians, and participants were aware of the assigned randomization group.

### Intervention Group (PW-PAD)

After randomization, all PW-PAD participants received the PAD document from research assistants. The PAD documents included future treatment and support preferences, early signs of relapse, and coping strategies. The research assistant proposed organizing the meeting with the peer worker and distributed the contact details. Depending on their preferences, the PW-PAD participants could:

- Meet a peer worker in a place of their choice.
- Be supported by this peer worker in drafting the PAD document with as many meetings as necessary. At this stage, the peer workers encouraged the sharing of PADs.
- Be supported by the peer worker during the sharing of PADs with the health care agent and the psychiatrist.

The PADs were completed and signed in paper format. They were stored by the health care agent or the psychiatrist, depending on the choice of the participant, and uploaded to electronic medical records if available and requested. In case of crisis, the existence of a PAD was reported by the patient, their companions, or informed caregivers.

### Control Group

People assigned to the control group were followed up as usual. They received comprehensive information about the PAD concept during the inclusion step and were free to complete a PAD. They were not introduced to a peer worker from the study.

### Outcomes

We collected data from the computerized patient administrative system and through participant interviews, which were scheduled every 6 months. The primary outcome was the rate of compulsory admissions to a psychiatric hospital at 12 months of follow-up, calculated as the number of participants with at least 1 compulsory admission divided by the number of participants.

Secondary outcomes were care-related, patient-reported, and mental health outcomes. The care-related outcomes included overall hospital admission rate (including voluntary and involuntary admissions), total number of admissions per patient (including voluntary and involuntary admissions), and rate of noncompulsory admissions per patient (ie, the proportion of total admissions per patient that was noncompulsory). Therapeutic alliance was assessed using the 4-point ordinal Alliance Scale.^19^ Higher scores indicate higher therapeutic alliance.

Among the patient-reported outcomes, quality of life was assessed using the Schizophrenia Quality-of-Life scale.^20^ Dimension and index scores range from 0, which indicates the lowest quality of life, to 100, which indicates the highest quality of life. Health status was assessed using the EuroQol scale (5 dimensions and 3 Likert).^21^ The index score ranges from 0, which indicates the worst health, to 1, which indicates the best health.

Mental-health outcomes included symptomatology assessed with the self-reported modified Colorado Symptom Index.^22^ Higher scores indicate a greater likelihood of mental health problems. Empowerment was assessed using the Empowerment Scale.^23^ The index scores are 0 to 100, where higher scores correspond to higher empowerment. Recovery was assessed using the Recovery Assessment Scale. A higher score indicates better recovery.

Other individual data collected at baseline included sociodemographic information and clinical data. Gender, age, education level, nationality, social benefits, wages, employment status, and housing conditions were collected. Deprivation was assessed using the EPICES score (English description of score name: Evaluation of Deprivation and Inequalities in Health Examination Centers).^24^ Clinical data assessed by the psychiatrist included somatic and addictive comorbidity and overall condition using the Clinical Global Impression scale, scored from 1 (healthy, not ill) to 7 (severely ill). Secondary outcomes are described in more detail in the eMethods in Supplement 1.

### Sample Size

The sample size was calculated to detect a reduction of 30% in the rate of compulsory admissions to psychiatric hospitals during the follow-up period of 12 months between the 2 groups^12,25,26^ with a reference point of 42.6%.^27^ To obtain a significance level of 2.5% and power of 80% with equal allocation to 2 groups, each group of the trial required 182 people. To allow for a potential 10% of people being lost to follow-up, the planned sample was 200 per group, for a total of 400.

### Statistical Analysis

The intention-to-treat analysis included all randomized participants. Data on compulsory admissions were obtained for all participants; no imputation was performed for the primary outcome. For secondary outcomes, missing data due to withdrawal, loss to follow-up, or nonresponse to specific items were 10% to 37.2% at 12 months. Missing data were addressed using multiple imputations,^28^ which creates multiple “complete” data sets with predictions for each missing value. This procedure takes into account uncertainty and yields accurate standard errors.^29^ Fifty imputed data sets were implemented using MICE by chained equations and mitools R packages. The multiple imputation approach was compared with existing methods for handing missing data; for complete cases, imputed data were compared with the mean or the last observation carried forward.

Data analysis was conducted in 3 steps. First, we performed a collinearity test on potential confounding factors based on unbalanced baseline characteristics. No collinearity was observed; the variance inflation factor ranged from 1.041 to 1.112. Second, the proportion of patients with compulsory admission was compared between groups using generalized estimating equations (GENLIN function), applying a binomial distribution with a link logit and adjusting for unbalanced baseline covariates (age, diagnosis, and Clinical Global Impression score), as well as site and site × group interaction. No group × covariate interactions were kept in the model (they were nonsignificant). Further, the subsequent model applied a logistic regression that provided a high goodness of fit. Adjusted odds ratios and risk differences with 95% CI were calculated.

For secondary outcomes, between-group differences were estimated using generalized estimating equations (GENLIN function), applying a normal distribution with a link identity for score variables or Poisson distribution with a link log for count variables. The β coefficient and effect sizes (Cohen *d*) with 95% CI were calculated. Statistical analysis was performed using SPSS 12 for Windows and RStudio version 3.2.1.

## Results

### Participants

As depicted in the Figure, 401 patients were randomized in total, of whom 7 (1.7%) were excluded from the study by the data board (4 patients allocated to the PW-PAD group and 3 patients allocated to the control group). Two were excluded because of noneligible inclusion criteria and 4 because they withdrew before any data collection. Of the 394 patients included in the study, 196 were assigned to the intervention group, and 198 were assigned to the control group (Figure). Interviews at the 12-month follow-up were completed for 127 (65%) in the PW-PAD group and 139 (70%) in the control group.

**Figure.**
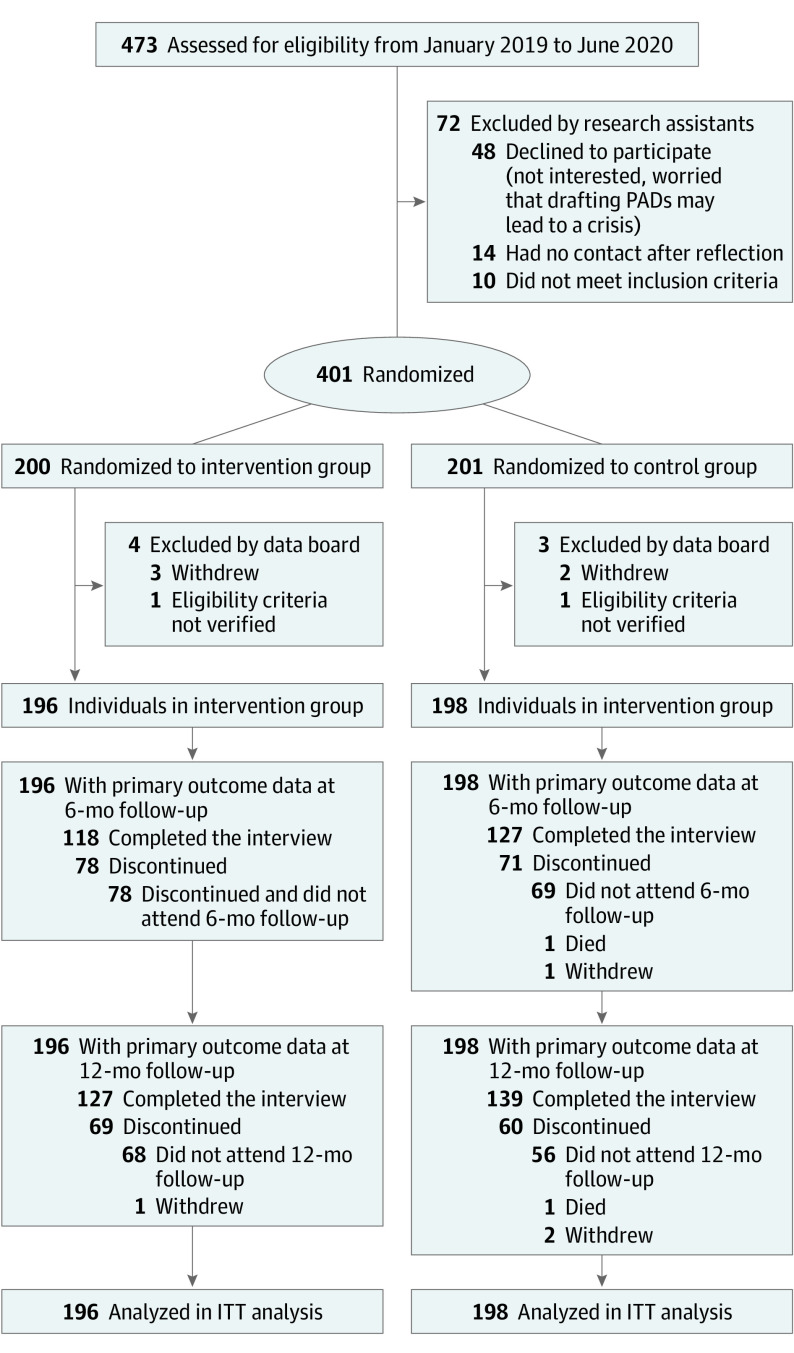
CONSORT Flow Diagram for the DAiP Trial (Peer Worker–Facilitated Psychiatric Advance Directive Study) ITT indicates intention to treat; PADs, psychiatric advance directives.

Baseline characteristics were similar between groups, except for age and severity (Table 1). Most patients were male (239, 60.7%) and had completed postsecondary school education (261, 66.4%). Of the 3 diagnoses assessed, 139 participants (36%) had bipolar I disorder, 178 (45%) schizophrenia, and 76 (19%) schizoaffective disorder. The median (IQR) age of the sample was 39 (29-48) years, and participants in the intervention group were younger (mean [SD], 37.4 [11.7] years vs 41.0 [12.7] years; *P* = .003). Baseline characteristics per site are provided in eTable 1 in Supplement 1. Characteristics at baseline were compared between complete and incomplete cases at the 12-month follow-up; significant differences were found in EPICES score, comorbidities, and previous hospital admissions (eTable 2 in Supplement 1).

**Table 1.** Sociodemographic and Clinical Characteristics of Participants (N = 394)

Characteristic	Group, No. (%)	
	PW-PAD (n = 196)	Control (n = 198)
Men	127 (64.8)	112 (56.6)
Women	69 (35.2)	86 (43.4)
Age, y		
Mean (SD)	37.4 (11.7)	41.0 (12.7)
Median (IQR)	36 (28-44)	40 (31-49)
French nationality	184 (93.9)	180 (91.8)
Education		
Less than HS	57 (29.2)	75 (37.9)
Completed HS or postsecondary school	138 (70.8)	123 (62.1)
Marital status		
Single	132 (67.3)	128 (64.6)
Married/partnered	38 (19.4)	35 (17.7)
Divorced/separated/widowed	26 (13.3)	35 (17.7)
Employed	33 (18.8)	37 (19.9)
EPICES score		
Mean (SD)	40.6 (19.9)	42.8 (20.9)
Median (IQR)	40.8 (24-57)	44.6 (26-59)
*DSM-5* diagnosis		
Schizophrenia	86 (44.1)	92 (46.5)
Bipolar I disorder	66 (33.8)	73 (36.9)
Schizoaffective disorder	43 (22.1)	33 (16.7)
Alcohol dependence	6 (3.4)	6 (3.5)
Substance dependence	22 (12.6)	24 (13.6)
≥1 Somatic comorbidity	120 (61.2)	137 (69.2)
CGI score		
Mean (SD)	4.1 (1.2)	4.3 (1.1)
Median (IQR)	4.0 (3-5)	4.0 (4-5)
No. of admissions in previous 1 y, mean (SD)	1.5 (0.9)	1.4 (0.8)
Patients with admissions in previous 1 y, No. (%)		
1 Admission	132 (67.3)	148 (75.5)
2 Admissions	45 (23.0)	37 (18.9)
≥3 Admissions	17 (8.7)	11 (5.6)

Abbreviations: CGI, Clinical Global Impression scale; EPICES, Evaluation of Deprivation and Inequalities in Health Examination Centers (English description); HS, high school; PW-PAD, peer worker–facilitated psychiatric advance directive.

Sixty-three participants in the intervention group (31.2%) and 67 in the control group (33.8%) were included during a hospitalization, with no significant differences in median (IQR) number of inpatient days between inclusion and discharge (34 [7-76] days vs 36 [11-67] days; *P* = .73).

### Completion of PADs

In the PW-PAD group, 107 participants completed a PAD document (54.6%) compared with 14 (7.1%) in the control group (*P* < .001). Among those, 81 met facilitators (75.7%), and 29 used PADs during a crisis in the 12-month follow-up period (27.1%) (Table 2).

**Table 2.** Outcomes at 12 Months Regarding Psychiatric Advance Directives for All Participants (N = 394)

Outcome	PW-PAD group (n = 196)	Control group (n = 198)	Total (N = 394)
Completion of PAD, No. (%)	107 (54.6)	14 (7.1)	121 (30.7)
Written with peer-worker support, No. (%)	81 (41.3)	4 (2.0)	85 (21.6)
% Among those who completed PAD in intervention group (n = 107)	75.7	NA	NA
Use of PAD during subsequent crisis, No. (%)	29 (14.8)	5 (2.5)	34 (8.6)
% Among those who completed PAD in intervention group (n = 107)	27.1	NA	NA
Compliance with PAD, No. (%)	22 (11.2)	5 (2.5)	27 (6.8)
% Among those who completed PAD in intervention group (n = 107)	20.6	NA	NA

Abbreviations: NA, not applicable; PAD, psychiatric advance directive; PW-PAD, peer worker–facilitated psychiatric advance directive.

### Primary Outcome

Table 3 presents the number and percentage of patients who had compulsory admissions to the hospital during the 12-month follow-up. The rate of compulsory admission was significantly lower in the PW-PAD group than in the control group: 27.0% (53 patients) vs 39.9% (79 patients), respectively (adjusted odds ratio, 0.58; 95% CI, 0.37 to 0.92; risk difference, −0.13; 95% CI, −0.22 to −0.04; *P* = .007).

**Table 3.** Compulsory Admissions, Overall Psychiatric Admissions, and Secondary Outcomes: Regression-Model Results at 12 Months Between Participants in the PW-PAD Group and Control Group

	No. (%) or mean (SD)		Logistic regression or GLM models		Effect size	
	PW-PAD group (n = 196)	Control group (n = 198)	β Coefficient (95% CI)	aOR (95% CI)^a^	Risk difference (95% CI)^b^	Cohen *d* (95% CI)^c^
**Primary outcome**						
Patients with ≥1 psychiatric compulsory admission, No. (%)	53 (27.00)	79 (39.90)	−0.57 (−1.01 to −0.08)^d^	0.58 (0.37 to 0.92)^d^	−0.13 (−0.22 to −0.04)^d^	NA
**Secondary outcomes**						
Patients with ≥1 psychiatric admission, No. (%)	70 (35.70)	79 (39.90)	0.15 (−0.29 to 0.59)	1.16 (0.75 to 1.80)	−0.04 (−0.13 to 0.54)	NA
No. psychiatric admissions per patient, mean (SD)	0.93 (2.19)	1.09 (2.02)	−0.16 (−0.64 to 0.25)	NA	NA	−0.08 (−0.30 to 0.12)
Rate of noncompulsory admissions per patient, mean (SD)	0.56 (0.45)	0.45 (0.45)	0.21 (−0.07 to 0.50)	NA	NA	0.47 (−0.17 to 1.11)
Score for each scale, mean (SD)						
4-PAS	35.62 (10.88)	31.56 (9.34)	1.83 (−0.35 to 4.13)	NA	NA	0.19 (−0.03 to 0.41)
S-QOL	62.39 (21.64)	57.62 (18.73)	3.77 (−0.39 to 7.94)	NA	NA	0.18 (−0.02 to 0.39)
EQ5D-3L	0.82 (0.27)	0.76 (0.32)	0.03 (−0.01 to 0.06)	NA	NA	0.17 (−1.08 to 14.0)
MCSI	11.49 (11.91)	13.87 (10.99)	−2.38 (−4.59 to −0.18)^d^	NA	NA	−0.20 (−0.40 to 0.00)^d^
ES	16.80 (26.32)	10.20 (16.04)	6.05 (1.56 to 10.53)^d^	NA	NA	0.30 (0.10 to 0.50)^d^
RAS	72.60 (14.13)	65.55 (13.92)	6.26 (3.29 to 9.23)^d^	NA	NA	0.44 (0.24 to 0.65)^d^

Abbreviations: 4-PAS, 4-point ordinal Alliance Scale; aOR, adjusted odds ratio; EQ5D-3L, EuroQol scale at 5 dimensions and 3 Likert; ES, Empowerment Scale; GLM, generalized linear model; MCSI, modified Colorado Symptom Index; NA, not applicable; PW-PAD, peer worker–facilitated psychiatric advance directive; RAS, Recovery Assessment Scale; S-QOL, Schizophrenia Quality of Life.

^a^ Logistic regression adjusting for age, diagnosis, Clinical Global Impression score, and site (site × group interaction was tested in GLM models and did not achieve statistical significance). High goodness of fit: Akaike information criterion, 436.6, compared with quasi-likelihood independence model criteria in GLM, 496.3. Adjusted odds ratios were reported for group variable.

^b^ Effect sizes were estimated from the difference in proportions and referred to the risk difference (with 95% CI computed).

^c^ Generalized linear models (using either a binomial distribution with a link logit, a negative normal distribution with a link log, or a Poisson distribution with a link log for count variables) adjusting for age, diagnosis, Clinical Global Impression score, and site and group × covariate interaction. A random effect (site) was fitted in the model with an exchangeable covariance matrix. No interactions were kept because none achieved statistical significance. The effect sizes were estimated from the mean difference divided by the pooled SD (using Cohen *d* formula) and based on the imputed-analysis set.

^d^ Statistically significant difference from the group variable (PW-PAD vs control groups).

### Secondary Outcomes

Secondary outcomes are presented in Table 3. Participants in the PW-PAD group exhibited lower symptoms as measured by modified Colorado Symptom Index score (effect size, −0.20; 95% CI, −0.40 to 0.00; *P* = .03), greater empowerment through Empowerment Scale scores (0.30; 95% CI, 0.10 to 0.50; *P* = .003), and higher recovery through Recovery Assessment Scale scores (0.44; 95% CI, 0.24 to 0.65; *P* < .001), compared with the control group. We found no statistically significant differences between groups for overall admission rate, therapeutic alliance, and quality-of-life measures.

Sensitivity analyses showed that the use of multiple imputations rather than other methods of handling missing data had little effect on the results (eTable 3 in Supplement 1).

## Discussion

Among 394 participants living with schizophrenia, bipolar I disorder, or schizoaffective disorder who had compulsory hospital admissions during the past year, use of PADs facilitated by peer workers was associated with a significant decrease in compulsory admissions and an increase in mental health outcomes (self-perceived symptoms, empowerment, and recovery) at 12 months. In the PW-PAD group, 54.6% of participants completed PADs (vs 7.1% in control group), among whom 75.7% used the support of peer workers.

This study is the first to our knowledge to show that PADs facilitated by peer workers are effective in reducing compulsory admissions. With a decrease of 32% of compulsory admissions, these results exceed the 25% pooled estimates from the meta-analysis. As in all other comparable studies, we found little effect on overall admissions, which supported that PADs might reduce compulsory admissions by making participants more willing to consider a voluntary admission when a crisis occurs instead of preventing hospital admissions.^5^ This result is important because minimizing compulsory admissions reduces its many widely described negative consequences.^30,31,32^ The qualitative research conducted in parallel with this study will provide insight into the mechanisms and drivers of the intervention’s effectiveness.

We found high rates of involuntary hospitalizations in both groups, which are consistent with published French data.^27^ France ranked above the international median calculated in 2017 among 22 countries, with an annual rate of 140.0 involuntary hospitalizations per 100 000 people compared with a median rate of 106.4 (IQR, 58.5-150.9).^33^

No previous RCTs on the efficacy of advance statements reported results of mental health outcomes, except Papageorgiou et al^11^ and Lay et al,^34^ who did not find any differences in psychiatric symptoms and psychiatric functioning at 12 months. In our study, PW-PAD was associated with improvement in mental health outcomes, with improvements in symptoms, empowerment, and recovery. Research on recovery-oriented services distinguishes between health-related outcome measures (such as symptoms) and recovery-oriented outcome measures (such as self-assessment of recovery, empowerment, or quality of life), which capture the efficacy of peer support more accurately.^35^ Research on peer support demonstrates substantial heterogeneity in terms of quality^36^ and encompasses many activities,^37^ but reviews have shown that the involvement of peers in various services is associated with mixed and limited improvements in recovery-oriented outcomes.^37,38,39^ Because the PW-PAD intervention had the greatest effect on several of these indicators, we hypothesize that peer-worker involvement plays a role in these results. Further studies should directly compare facilitation by health care professionals and facilitation by peer workers using a measurement of perceived coercion.

Furthermore, the high rates of completion in the PW-PAD group show the importance of encouragement (explanation, distribution of the document) and facilitation, which reinforces previous research.^5,6,14^

### Strengths and Limitations

Our study has 2 major strengths. First, the nature of this research is highly participatory because it involved patients at all levels from the beginning.^16^ Second, it was deployed through more than 40 psychiatrists from all backgrounds with current practices. Because peer workers were independently recruited by research teams, participating units were not only recovery-oriented but reflected a variety of services. Thus, the study suggests that PW-PADs can be easily implemented and our results are generalizable to other services.

This trial had several limitations. First, the follow-up was complicated by the COVID-19 health crisis, and the loss of 31% of participants at the 12-month follow-up and resulting decrease in power for secondary outcomes are important limitations. Fortunately, our primary outcome was based on administrative data and consequently not affected. Second, the recruitment was unequal among centers, and we did not have the power to make comparisons across the 7 different centers. Third, the profession of peer worker is relatively new in France, and the PAD was a new tool. The study has led to the development of specific training for peer workers and teams, which should help improve the results of such interventions. Further, we had notably restrictive criteria, and the findings may not be generalizable to other psychiatric populations.

## Conclusions

Among people living with schizophrenia, bipolar I disorder, or schizoaffective disorder, the use of PW-PADs was associated with a significant decrease in compulsory admissions and a significant increase in some mental health outcomes (self-perceived symptoms, empowerment, and recovery). These findings support the use of PW-PADs for people with schizophrenia, bipolar I disorder, or schizoaffective disorder. Legal and organizational initiatives that promote supported decision-making can develop the activity of peer workers to fulfill this mission.

## Notes

Supplement 1. **eTable 1.** Sociodemographic and clinical characteristics of participants according to sites **eTable 2.** Comparison of complete cases at M12 (n=266) and incomplete cases (n=128) with baseline characteristics and secondary outcomes at 6 months **eTable 3.** Results of descriptive statistics of secondary outcomes according to different approaches to handling missing data **eMethods.** Secondary outcome measures

Supplement 2. Trial protocol and statistical analysis plan

Supplement 3. Collaborators in the DAiP group

Supplement 4. Data sharing statement
