# Supplementary material for: Effect of Psychiatric Advance Directives Facilitated by Peer Workers on Compulsory Admission Among People With Mental Illness: A Randomized Clinical Trial
Source: JAMA Psychiatry. 2022 Jun 6;79(8):752–9. doi: 10.1001/jamapsychiatry.2022.1627 (PMC9171654; doi:10.1001/jamapsychiatry.2022.1627)
Supplement: Supplement 1. — eTable 1. Sociodemographic and clinical characteristics of participants according to sites eTable 2. Comparison of complete cases at M12 (n=266) and incomplete cases (n=128) with baseline characteristics and secondary outcomes at 6 months eTable 3. Results of descriptive statistics of secondary outcomes according to different approaches to handling missing data eMethods. Secondary outcome measures [file jamapsychiatry-e221627-s001.pdf]

Supplemental Online Content

Tinland A, Loubière S, Mougeot F, et al; DAiP Group. Effect of psychiatric advance directives facilitated by peer workers on compulsory admission among people with mental illness: a randomized clinical trial. *JAMA Psychiatry*. Published online June 6, 2022. doi:10.1001/jamapsychiatry.2022.1627

eTable 1. Sociodemographic and clinical characteristics of participants according to sites

eTable 2. Comparison of complete cases at M12 (n=266) and incomplete cases (n=128) with baseline characteristics and secondary outcomes at 6 months

eTable 3. Results of descriptive statistics of secondary outcomes according to different approaches to handling missing data

eMethods. Secondary outcome measures

This supplemental material has been provided by the authors to give readers additional information about their work.

© 2022 Tinland et al. *JAMA Psychiatry*.

eTable 1. Sociodemographic and Clinical Characteristics of Participants according to sites

	Site 1 Marseille			Site 2 Paris			Site 3 Lyon			
**Baseline characteristics**	**Total (n=144)**	Centre 1 (n=137)	Centre 3 (n=7)	**Total (n=100)**	Centre 2 (n=16)	Centre 4 (n=84)	**Total (n=150)**	Centre 5 (n=19)	Centre 6 (n=8)	Centre 7 (n=123)
Pw-PAD Group, No. (%)	**71 (49.3)**	67 (48.9)	4 (57.1)	**50 (50.0)**	8 (50.0)	42 (50.0)	**75 (50.0)**	11 (57.9)	3 (37.5)	61 (49.6)
Male gender, No. (%)	**84 (58.3)**	78 (56.9)	6 (85.7)	**60 (60.0)**	11 (68.8)	49 (58.3)	**95 (63.3)**	13 (68.4)	3 (37.5)	79 (64.2)
Age mean (SD), y	**38.9 (12.4)**	38.9 (12.5)	38.1 (13.0)	**42.3 (13.5)**	37.5 (9.6)	43.1 (13.9)	**37.4 (11.1)**	36.9 (9.1)	42.0 (10.4)	37.2 (11.5)
French nationality, No. (%)	**138 (95.8)**	133 (97.1)	5 (71.4)	**82 (83.7)**	11 (78.6)	71 (84.5)	**144 (96.0)**	18 (94.7)	8 (100)	118 (95.9)
Completed or postsecondary school, No. (%)	**88 (61.1)**	86 (62.8)	2 (28.6)	**57 (57.6)**	9 (56.2)	48 (57.8)	**116 (77.3)**	14 (73.7)	4 (50.0)	98 (79.7)
Single marital status, No. (%)	**95 (66.0)**	89 (65.0)	6 (85.7)	**63 (63.0)**	8 (50.0)	55 (65.5)	**102 (68.0)**	13 (68.4)	5 (62.5)	84 (68.3)
Having work activity, No. (%)	**21 (15.4)**	21 (16.2)	0 (0)	**20 (24.4)**	2 (13.3)	18 (26.9)	**29 (20.1)**	3 (16.7)	1 (12.5)	25 (21.2)
EPICES score mean (SD)	**42.3 (20.4)**	42.3 (20.7)	42.0 (15.4)	**54.5 (18.2)**	50.6 (15.3)	55.2 (18.7)	**33.7 (17.5)**	29.2 (16.3)	40.5 (23.2)	33.9 (17.3)
DSM-5 diagnosis, No. (%)										
Bipolar I disorder	**82 (56.9)**	81 (59.1)	1 (14.3)	**19 (19.2)**	1 (6.2)	18 (21.7)	**28 (25.3)**	10 (52.6)	2 (25.0)	26 (21.1)
Schizophrenia	**45 (31.2)**	40 (29.2)	5 (71.4)	**51 (51.5)**	10 (62.5)	41 (49.4)	**82 (54.7)**	8 (42.1)	6 (75.0)	68 (55.3)
Schizoaffective disorders	**17 (11.8)**	16 (11.7)	1 (14.3)	**29 (29.3)**	5 (31.2)	24 (28.9)	**30 (20.0)**	1 (5.3)	0 (0)	29 (23.6)
Alcohol dependence, No. (%)	**2 (1.7)**	2 (1.8)	0 (0)	**5 (6.1)**	0 (0)	5 (6.8)	**5 (3.4)**	2 (10.5)	1 (12.5)	2 (1.7)
Substance dependence, No. (%)	**23 (18.9)**	23 (19.8)	0 (0)	**10 (12.2)**	0 (0)	10 (13.5)	**13 (8.8)**	3 (16.7)	3 (37.5)	7 (5.8)
Having comorbidities, No. (%)	**89 (61.8)**	87 (63.5)	2 (28.6)	**43 (43.0)**	6 (37.5)	37 (44.0)	**125 (83.3)**	16 (84.2)	8 (100)	101 (82.1)
CGI Score, mean (SD)	**4.1 (1.4)**	4.1 (1.4)	3.5 (2.0)	**4.7 (1.2)**	4.4 (1.3)	4.7 (1.2)	**4.0 (0.9)**	3.8 (0.8)	3.9 (0.8)	4.0 (0.9)
Number of admissions 1 year ago >=3, No. (%)	**7 (4.9)**	7 (5.1)	0 (0)	**10 (10.2)**	2 (12.5)	8 (9.8)	**11 (7.3)**	1 (5.3)	2 (25.0)	8 (6.5)

Pw-PAD: peer-worker facilitated psychiatric advance directive; SD: standard deviation; IQR: interquartile range; DSM-5: Diagnostic and Statistical Manual of Mental Disorders, fifth edition; CGI: Clinical Global Impression scale.

© 2022 Tinland et al. *JAMA Psychiatry*.

eTable 2. Comparison of complete cases at M12 (n=266) and incomplete cases (n=128) with baseline characteristics and secondary outcomes at 6 months

**Baseline characteristics**	Complete cases (n=266)	Incomplete cases (n=128)	P-value
Study Group No. (%)			
Pw-PAD Group	127 (47.7)	69 (53.9)	0.252
Control Group	139 (52.3)	59 (46.1)	
Gender, No. (%)			
Men	153 (57.5)	86 (67.2)	0.066
Age mean (SD), y	39.7 (12.8)	38.1 (12.6)	0.234
Nationality, No. (%)			
French	249 (94.0)	115 (90.6)	0.220
Education, No. (%)			
Completed or postsecondary school	181 (68.0)	80 (63.0)	0.321
Marital status, No. (%)			
Single	173 (65.0)	87 (68.0)	0.822
Married/partnered	50 (18.8)	23 (18.0)	
Divorced/separated/widow	43 (16.2)	18 (14.1)	
Work activity, No. (%)			
Yes	45 (18.0)	25 (22.3)	0.336
EPICES score mean (SD)	40.2 (19.6)	44.8 (21.7)	0.039
DSM-5 diagnosis, No. (%)			
Bipolar I disorder	98 (37.0)	41 (32.0)	0.528
Schizophrenia	115 (43.4)	63 (49.2)	
Schizoaffective disorders	52 (19.6)	24 (18.8)	
Alcohol dependence, No. (%)			
Yes	9 (3.7)	3 (2.8)	0.650
Substance dependence, No. (%)			
Yes	31 (12.8)	15 (13.9)	0.772
Having comorbidities, No. (%)			
Yes	184 (69.2)	73 (57.0)	0.018
CGI Score, mean (SD)	4.1 (1.2)	4.4 (1.2)	0.048
Number of admissions in previous 1 year, mean, No. (%)			
1	187 (70.6)	93 (73.2)	0.043
2	51 (19.2)	31 (24.4)	
>=3	11 (9.4)	3 (2.4)	
**6-month secondary outcomes**			
4-PAS, mean (SE)	34.7 (6.6)	35.5 (6.8)	0.552
S-QOL Score, mean (SD)	58.3 (16.1)	63.1 (13.4)	0.079
EQ5D score, mean (SD)	0.78 (0.2)	0.79 (0.2)	0.613
MCSI score, mean (SD)	13.5 (9.2)	12.0 (9.6)	0.319
ES score, mean (SD)	12.5 (16.0)	10.4 (15.0)	0.445
RAS score, mean (SD)	67.3 (12.2)	69.1 (11.6)	0.384

© 2022 Tinland et al. *JAMA Psychiatry*.

Pw-PAD: peer-worker facilitated psychiatric advance directive; SD: standard deviation; IQR: interquartile range; DSM-5: Diagnostic and Statistical Manual of Mental Disorders, fifth edition; CGI: Clinical Global Impression scale.

© 2022 Tinland et al. *JAMA Psychiatry*.

eTable 3: Results of descriptive statistics of secondary outcomes according to different approaches to handling missing data

Secondary outcomes	Type of analysis	Pw-PAD group	Control group
4-PAS score			
	Primary analysis on imputed data (n=394)£	35.62 (7.59)	33.56 (6.76)
	Complete cases analysis (N=266)§	35.81 (7.22)	33.56 (6.43)
	Analysis on imputed data using mean %	35.81 (4.77)	35.56 (4.69)
	Analysis on imputed data using "Last observation carried forward" (LOCF) &	35.04 (7.03)	33.90 (6.69)
S-QOL Score			
	Primary analysis on imputed data (n=394)£	62.39 (17.01)	57.62 (14.88)
	Complete cases analysis (N=266)§	63.17 (16.01)	58.00 (14.91)
	Analysis on imputed data using mean %	63.17 (11.75)	58.00 (11.39)
	Analysis on imputed data using "Last observation carried forward" (LOCF) &	61.59 (15.68)	57.72 (15.35)
EQ5D score			
	Primary analysis on imputed data (n=394)£	0.82 (0.21)	0.76 (0.27)
	Complete cases analysis (N=266)§	0.82 (0.21)	0.76 (0.25)
	Analysis on imputed data using mean %	0.82 (0.17)	0.76 (0.21)
	Analysis on imputed data using "Last observation carried forward" (LOCF) &	0.82 (0.21)	0.76 (0.27)
RAS Score			
	Primary analysis on imputed data (n=394)£	72.60 (11.30)	65.55 (11.79)
	Complete cases analysis (N=266)§	72.57 (12.07)	65.69 (11.32)
	Analysis on imputed data using mean %	72.57 (9.47)	65.69 (9.33)
	Analysis on imputed data using "Last observation carried forward" (LOCF) &	71.25 (12.44)	66.93 (12.49)
ES Score			
	Primary analysis on imputed data (n=394)£	16.80 (19.38)	10.20 (12.37)
	Complete cases analysis (N=266)§	16.76 (19.56)	10.06 (12.30)
	Analysis on imputed data using mean %	16.76 (15.01)	10.06 (9.84)
	Analysis on imputed data using "Last observation carried forward" (LOCF) &	15.55 (18.76)	11.64 (16.55)
MCSI Score			
	Primary analysis on imputed data (n=394)£	11.49 (9.07)	13.87 (8.71)
	Complete cases analysis (N=266)§	11.37 (9.21)	13.97 (9.11)
	Analysis on imputed data using mean %	11.37 (7.28)	13.97 (7.46)
	Analysis on imputed data using "Last observation carried forward" (LOCF) &	11.68 (9.02)	13.88 (9.27)

© 2022 Tinland et al. *JAMA Psychiatry*.

[£]: Based on multiple imputation approach; the data set included all randomized individuals at baseline (n=394).
[§]: Based on complete cases; the data set included all randomized individuals at baseline who completed 12-month follow-up (n=266).
[%]: Based on imputation approach by the mean in each group; the data set included all randomized individuals at baseline (n=394).
[&]: Based on imputation approach by the last observation carried forward; the data set included all randomized individuals at baseline (n=394).
[@]: Based on imputation approach using a worse-case scenario; in this approach, the worst value observed in the complete cases at 12-month follow-up in the intervention group was attributed to missing cases in the intervention group, the best value observed in the complete cases at 12-month follow-up in the control group was attributed to missing cases in the control group; the data set included all randomized individuals at baseline (n=394).

© 2022 Tinland et al. *JAMA Psychiatry*.

eMethods: Secondary outcome measures

- Care-related outcomes:
    - Overall hospital admission rate (including voluntary and involuntary admissions)
    - Overall hospital admission rate (including voluntary and involuntary admissions)
    - Total number of admissions per patient (including voluntary and involuntary admissions).
    - Rate of non-compulsory admissions per patient, i.e., the proportion of total admissions per patient which was non-compulsory.
    - Therapeutic alliance assessed using the 4-Point ordinal Alliance Scale (4-PAS) [17]. This self-rating scale includes two dimensions (empathy experienced and psychoeducation) and a global score. Higher scores indicate higher therapeutic alliance.
- Patient-reported outcomes:
    - Quality of life assessed using the Schizophrenia Quality-of-Life scale (S-QoL 18 scale), which comprises 18 items evaluating eight dimensions: psychological well-being, self-esteem, family relationships, relationships with friends, resilience, physical well-being, autonomy, and sentimental life[1] [18]. Dimension and index scores range from 0, indicating the lowest quality of life, to 100, the highest quality of life. S-QoL-18 has been also validated in bipolar disorders.
    - Health status is assessed using the EQ5D-3L [19], a standardized non-disease-specific instrument for describing and valuing health states, throughout five dimensions: mobility, personal care, routine occupations, pain and discomfort, and anxiety and depression. Each dimension has three response levels of severity. Index score ranges from 0, indicating the worst health, to 1 the best health.
- Mental-health outcomes:
    - Mental health symptomatology assessed using the self-report Modified Colorado Symptom Index (MCSI) [20]. This 14-item tool evaluates how often in the past month

[1] I. e. Emotional life

© 2022 Tinland et al. *JAMA Psychiatry*.

an individual has experienced a variety of mental health symptoms, including loneliness, depression, anxiety, and paranoia. Higher scores indicate a greater likelihood of mental health problems.

- Empowerment assessed using the Empowerment Scale (ES) [21] which is a specific tool for mental health. It comprises five dimensions: self-esteem, optimism, power, activism, and legitimate anger. Index scores range from 0 to 100, with higher scores corresponding to higher empowerment.
- Recovery assessed using the Recovery Assessment Scale (RAS), which measures various aspects of recovery from the consumer's point of view [22,23] This self-administered instrument comprises 24 items, exploring five domains: personal confidence and hope, willingness to ask for help, goal and success orientation, reliance on others, and not being dominated by symptoms. A higher score indicates better recovery.

© 2022 Tinland et al. *JAMA Psychiatry*.
