# Supplementary material for: Effect of Psychiatric Advance Directives Facilitated by Peer Workers on Compulsory Admission Among People With Mental Illness: A Randomized Clinical Trial
Source: JAMA Psychiatry. 2022 Jun 6;79(8):752–9. doi: 10.1001/jamapsychiatry.2022.1627 (PMC9171654; doi:10.1001/jamapsychiatry.2022.1627)
Supplement: Supplement 2. — Trial protocol and statistical analysis plan [file jamapsychiatry-e221627-s002.pdf]

An interventional multicenter, randomized, comparative, prospective trial assessing the impact on the health care pathway of Psychiatric Advance Directives facilitated by peer-workers (pw-PAD) for people suffering from schizophrenia, bipolar-I disorders or schizoaffective disorders. DAiP study

Research program on health care pathway performance
PREPS 2017 – 0575

Research protocol – English Version (trad. Justine BUAND)
Version integrating the Statistical Analysis Plan

Investigator coordinator: Dr Aurélie TINLAND
Pôle psychiatrique universitaire Solaris, hôpital Sainte-Marguerite, APHM, 261 Bd Sainte Marguerite, 13009 Marseille.
EA 3279 : CEReSS - Centre d'Etude et de Recherche sur les Services de Santé et la Qualité de vie, Aix-Marseille Université, Faculté de Médecine - Secteur Timone, 27 bd Jean Moulin, 13005 Marseille

Sponsor: Assistance Publique Hôpitaux de Marseille
80 rue Brochier, 13354 Marseille

Research Team:

Pr. Pascal Auquier; Dr. Magali Pontier; Mrs. Léa Leclerc; Mrs. Sandrine Loubière; Mr. Benoît Eyraud; Mr. Julien Grard; Mrs. Magali Coldefy; Dr. Edouard Leaune; Dr. Patrick Lecardinal; Dr. Jacques Glikma; Dr Nathalie Christodoulou; Dr. Sophie Cervello; Mr. Tim Greacen; Pr. Nicolas Franck; Dr. Vincent Girard; Dr. Karine Baumstark ; Pr. Christophe Lançon; Pr. Jean Naudin; Pr. Pierre Le Coz; Dr. Guillaume Fond.

VERSIONS

VERSIONS	Object and date of request	Date	Advice of the French ethics committee (Comité de protection des personnes du Sud-Ouest et Outre-Mer 4)	Date
**V1**	**Initial submission**	29 May 2018	Request for minor changes	28 June 2018
**V2**	**Revised submission**	24 July 2018	Avis favorable (Positive statement)	31 August 2018
**V3**	**Opening of new recruitment sites with new investigators: Valvert and Edouard Toulouse (Marseille), Saint Jean de Dieu (Lyon)**	8 April 2019	Avis favorable (Positive statement)	23 May 2019
**V4**	**Opening of new recruitment sites with new investigators Argenteuil (Paris)**	26 September 2019	Avis favorable (Positive statement)	18 October 2019

Table of contents

I. Introduction *6*
A. **Consent, care without consent: ethical and legal principles** 6
B. **Involuntary Hospital Admission** 7
C. **Psychiatric disorders and recovery** 7
D. **Reintroduction of consent in situations of decisional incompetence** 9
E. **Psychiatric Advance Directives** 9
1. Different forms of Psychiatric Advance Directives 10
2. Psychiatric Advance Directive Results 11
2.1 Quantitative PAD results 11
2.2 Qualitative PAD results 12
F. **Intervention in France** 12
II. Objectives *14*
A. **Quantitative Objectives** 14
1. Outcomes 14
2. Structures and processes 14
B. **Qualitative Objectives** 14
C. **Participatory Research Objectives** 14
III. Project steering and partners *16*
D. **Steering committee and scientific committee for the program** 16
1. Steering committee for experimentation 16
2. Scientific committee 16
E. **Assessment team** 16
1. Staff 17
2. Assessment steering committee 17
3. Users' committee 18
F. **"DaiP" project-bearing teams** 18
1. Lyon 18
2. Marseille 18
3. Paris 19
IV. Care strategies under study *19*
A. **Pw-PAD intervention** 19
1. Description of the contents and procedure 19
2. Description of the frequency 19
B. **Usual care** 20
1. Description of contents, frequency and process 20
V. Study population *20*
A. **Inclusion criteria** 20
B. **Exclusion criteria** 20
C. **Trial exit criteria** 20
VI. Methodology *21*
A. **Quantitative part of research** 21
1. Experimental design diagram 21
2. Judgment criteria and collected variables 21
3. Data collection periods 23
B. **Procedure** 23
1. Detection – Eligibility 24
2. Inclusion 24

3. Initial assessment ... 25
4. Follow-up ... 25
C. Number of subjects necessary and feasibility ... 26
1. Number of subjects necessary ... 26
2. Feasibility – Trial duration ... 26
D. Data processing ... 27
1. Data sources ... 27
2. Data quality control ... 27
3. Data entry ... 27
E. Statistical Plan Analysis (SAP) ... 27
1. Analysis Populations ... 27
2. Effectiveness Analysis ... 27
2.1. Primary Outcome Measures ... 27
2.2. Secondary Outcome Measures ... 28
3. Safety analyses ... 28
4. Sensitivity Analyses ... 28
5. Other analyses ... 29
6. Handling of Missing Data ... 29
7. Interim, Final Analyses and Timing of Analyses ... 29
F. Qualitative assessment ... 29
1. Challenge and presentation of the themes of investigation ... 30
2. Methodological tools ... 30
3. Summary table ... 35
4. Expectations: the question of generalizing the program ... 35
VII. Calendar ... 37
VIII. Consistency with regards to research policies, perspectives and feasibility ... 38
A. Consistency with regards to health policies ... 38
B. Expected results and perspectives ... 40
C. Feasibility ... 40
D. Limitation and justification ... 40
1. Experimental plan ... 40
2. Contagion of controls ... 41
3. Absence of pairing ... 41
IX. Ethical and regulatory aspects ... 41
A. Risk-benefit ratio ... 41
1. Benefits and advantages ... 41
2. Risks and constraints ... 42
3. Risk-benefit ratio ... 42
B. Legal and ethical aspects ... 42
1. Institutional review board ... 42
2. Data protection ... 43
3. Stopping the trial ... 44
4. Quality control and insurance ... 44
5. Data archiving ... 45
C. Clinical trials vigilance ... 45
X. Report and publications ... 45
XI. Valorization and societal sensitization ... 45
XII. References ... 45

XIII. Annexes *53*
A. Annex 1: French legal modes of care without consent in psychiatry since the act of July 5, 2011, amended September 27, 2013 .... **53**
B. Annex 2: International political and legal context **54**
Article 12 54
C. Annex 3: Act of February 2016 on advance directives {Article L.1111-11 of the Code de la Santé Publique (CSP; French public health regulations)} and healthcare agents {Article L.1111-6 of CSP} **56**
D. Annex 4: Human resources for the interventional research on each site **57**
E. Annex 5: Document "Directives Anticipées incitatives en Psychiatrie" "Psychiatric advance directives" developed by the organization Eutopia – (Document not recuperated by the coordinating center and for data analysis) **58**
F. Annex 6: Information sheet intended for professionals **62**

I. Introduction

A. Consent, care without consent: ethical and legal principles

"Respect for persons incorporates at least two ethical convictions: first, that individuals should be treated as autonomous agents, and second, that persons with diminished autonomy are entitled to protection. The **principle of respect for persons** thus divides into two separate moral requirements: the requirement to **acknowledge autonomy** and the requirement to **protect those with diminished autonomy**."
Belmont Report, 1979 (6)

In order to respect the principle of autonomy, **consent is indispensable** for any medical care (article L.1111-4 of the Code de la Santé publique (French public health regulations)), but, considering that a lack of psychiatric care can be detrimental to the patient, restrictions of the exercise of personal freedom, limited to those required by the state of health and the implementation of the therapy, are organized by law.

In France, **treatment without consent is governed by the act of July 5, 2011[1],** which replaced the act of June 27, 1990, with the wish to affirm the rights of people receiving psychiatric therapy: information, mandatory decision by the Juge des Libertés et de la Détention, diversification of the types of care with the possibility of ambulatory treatment without consent (7).
Several legal types of care without consent exist, for which there are **3 indispensable criteria: 1) the presence of mental disorders, 2) impossibility to consent to care, 3) the necessity for treatment and for constant or regular medical surveillance.**
The assessment of these criteria is placed under the responsibility of two different psychiatrists, or one in case of emergency (modality SDT-U), who draw up a medical certificate for admission based on their clinical findings.
A handwritten request for hospital admission by a "third party", i.e. a member of the person's family or someone susceptible of acting in the person's best interest must be joined to these certificates, except in cases of "imminent peril" (modality SDT-PI).
In case of "imminent danger for people's safety or public order", the request is issued by a State representative, generally the Préfet, by prefectoral decree (SDRE, which replaces the old modality of compulsory hospital admission).
The placement of a person against their will in an institution is reassessed at regular intervals to monitor the different criteria and justify whether the measure is continued or lifted.
Annex 1 details the different legal types of care without consent in France.

When it comes to stating the **capacities of individuals to decide and act by themselves**, like the psychiatrists do for care without consent, authorized people in fact have to consider whether these individuals are capable of behaving like autonomous individuals. They are thus faced with the difficulty of defining **autonomy**, with **vulnerability** and with the related moral and political philosophical issues. At what point can one say that these individuals are no longer supposed to be autonomous without risk? In other words, when does protection preserve this autonomy, and on the contrary when does it begin to restrict it?(8) Which legal and social conditions enable people to exercise their autonomy? (9,10)

Since the 1980's, Anglo-Saxon studies have proposed criteria **to assess decision-making capacity** concerning consent to healthcare(11), assessing several dimensions: understanding, appreciation, reasoning and/or expression of choice. A certain number of standardized tools were assessed in a convincing manner, some specific[2] to other generics, most presenting good psychometric properties (12).
In France, the assessment of legal capacity is not very procedural, is more clinical and centered on the "case" (13).

---

[1] Amended by the act of September 27, 2013
[2] Specific for psychiatric disorders, Alzheimer's disease or mental deficiency.

B. Involuntary Hospital Admission

Between 2012 and 2015, the practice of "involuntary treatment"[3] in connection with cases of psychiatric crisis situations increased by 15 % in France, i.e. a sharper increase than that of the active file for psychiatry, proportionately as well as in absolute value.
Approximately 92 000 people received involuntary care in 2015 (14). Among these, 79 000 were admitted to hospital (progression of 13%), making France one of the European countries that practices the most involuntary hospital admission. 16 000 people were placed in SDRE.
The average reported duration of medical programs (hospital and ambulatory) was between 12 and 22 months according to the legal modes, including 75 days in hospital (61 days without consent) (15).
During these involuntary hospital stays, the practice of seclusion, forbidden in other countries for ethical reasons (16), also increased: 8% of people involuntarily hospitalized in 2015 versus 4% in 2003 (14), which alerted organizations such as the French national observatory for places of deprivation of liberty, or the UN[4] (Annex 2.2).

Concerning **involuntary hospital admission**, there are important national (14), European (17) and international (18) **disparities**:

- Quantitatively: average rate 177 /100 000 inhabitants in France, varying with a 1:6 ratio depending on the geographic department; rates between 6 and 218 / 100 000 inhabitants in Europe depending on the country;
- Qualitatively between countries: variations in terms of procedures, legal criteria (notions of danger, health needs), of the authority making the decision for coercion (medical/non medical) etc.

This **frequent and wide-spread use,** admittedly disparate, of coercion does not seem to be based on its efficiency, a systemic literature review on treatment without consent effectively found **little proof of the efficiency of these measures** in terms of state of health, social functioning, healthcare service use or of satisfaction (19).
Negative effects of involuntary hospital admission were identified in several studies, showing that these periods of deprivation of liberty are a very negative experience for the patient and can cause damage (20,21) that they have a negative impact on quality of life (22) and finally that the avoidance of future hospital admission has a negative effect on prognosis.

The practice, or rather the variety of practices connected with coercion (23) is furthermore at the hub of **controversies** (24) and **ethical dilemmas** among psychiatrists (25). Some authors have reported the double "nominative fragility" with which they are faced: according to the most general social norms, they are trapped with the necessity of articulating autonomy and protection of the people they treat, without having evident guidelines or expert opinions at their disposal to help them act (26).

All types of hospitalization included, schizophrenic patients in particular have **very high rates of hospital readmission** (70.5% over 10 ans, 25% over 4 months) resulting in high medical costs (27). A randomized trial showed the inefficiency of coercion on the prevention of hospital readmission (28). A meta-analysis in 2017 assessed that 142 coercive measures were necessary to avoid 1 readmission (19).

C. Psychiatric disorders and recovery

In France, **half of the involuntary hospital admissions concern people suffering from schizophrenia, other psychoses or bipolar disorders** (14).

---

[3] " involuntary treatment" includes involuntary hospitalization and ambulatory treatment.
[4] Convention against torture and other penalties or cruel, inhumane or degrading treatments CAT/C/FRA/CO/7

Despite the fact that different countries use different diagnostic classifications, schizophrenia, schizoaffective disorders and mania (decompensation of bipolar-I disorder) are considered to represent between 50% (Belgium) and 70% (Netherlands) of involuntary admissions, with schizophrenia being the dominant pathology (17). Hallucinations, cognitive disorders, psychomotor agitation are the symptoms for which the intensity leads most often to involuntary hospital admission (18). These symptoms are those of psychotic disorders: schizophrenia, schizoaffective disorders, bipolar-I disorders (DSM-IV).

These psychiatric disorders are frequently characterized by an alternation of psychotic episodes and periods of stability (29) (30). Symptomatic fluctuation is accompanied by more or less severe cognitive alterations (31), and can thus affect capacity[5]. Data from available meta-analyses suggest a significant overlap of deficiencies in the neuropsychological profile of bipolar and schizophrenic patients, with differences that are more quantitative than qualitative. Cognitive deficiencies can be identified early in both pathologies, and appear during the first episode, whether manic or psychotic (32).

The notion that these disorders were incurable was maintained for over two centuries (33) despite epidemiological (34),clinical (35) and anthropological (36) data being much more optimistic. Indeed, after first-episode psychosis, 20% will never relapse, 50% will have other episodes alternating with long periods of remission, and 30% will have permanent symptoms. Furthermore, there is a specific difference for these diseases between clinical remission and the possibility for these individuals to have a social life(37).

It is the people suffering from psychotic disorders themselves who have led to a change of paradigm that takes these specificities into account: **the notion of recovery** that first appeared in the United States in the wake of the civil rights movement.

With this concept where the aim is not to make the symptoms disappear but to actually live a satisfying life despite the limits of one's disease, to restore the person's active posture to reengage in an active and social life is crucial, feeling that one has control of one's life again is a goal but also a way to encourage progress towards this goal. Recovery is described as being as biological as it is social and political (38).
The recovery rate for schizophrenia was estimated at over 60% (National Advisory Mental Health Council, 2007).

Studies on **recovery-oriented services** which have proved their efficiency (evidence-based psychiatry)(35) showed the importance of **backing the person's self-determination, empowerment** (restoring power to decide and act) and **autonomization**(39), which specifically echoes with current healthcare policy strategies in our country (see § VIII-A).

One of the innovations brought about by the "recovery" movement was the emergence of a new profession, "**peer-worker**" in the support teams. Patients or ex-patients in psychiatry, having had or still having mental disorders, recovered or en route for recovery, peer-workers are capable of using their personal experience of the disease and their own recovery pathway, as well as their knowledge of psychiatric services in order to create a different bond with the patients, a trusting and close relationship. Assessment of their interventions showed that they contributed to better treatment access (40) and they brought hope by being an incarnation of recovery (41).
In France, the peer-worker profession is still not widespread, despite several experiments and conclusive practices on several sites.

Nowadays recovery is the basis for mental health policies in numerous countries including Canada, the United States, new Zealand or Australia. Peer-workers are strongly involved at all levels.

---

[5] The capacity to give consent or to refuse treatment signifies the capacity to understand the aim, the type, the probable effects and the risks of the treatment in question, including its chances of success, the consequences of it not being given and of any other connected replacement option.

D. Reintroduction of consent in situations of decisional incompetence

How to encourage autonomy and the principle of consent in care? This question is also asked for other categories of vulnerable people such as people with intellectual deficiencies, or old people, especially with Alzheimer's disease.

The UN Convention on the Rights of Persons with Disabilities (CRPD) pleads in favor of a **system of "supported decision-making"** which would make it possible not to make decisions for someone else and to avoid the use of coercion. In their comments on article 12, the CRPD underlined that this implied a transition from "substituted" to "supported" decision-making (42) ; (43). In this framework, new judicial tools have been available for the last ten years or so with the aim to take the opinion of people more into account (ex. future protection mandate, life project, MASP).

Advance directives are set up by a person who still has decision-making capacities, but who is anticipating potential incapacity, potential decline of their functional capacities. Their goal is to give the opinion of a person who is no longer capable of expressing themselves due to incapacity, to **represent the expression of a patient's free will**. Thus, they support one of the key principles of medical ethics: the patient's autonomy (44).

In France, advance directives related to **end-of-life** care are a right stipulated in the Leonetti act of April 22, 2005 concerning the rights of the ill and of the end-of-life (45). Defined in article L1111-11 of the Code de la Santé Publique (French public health regulations), advance directives are written directives allowing any adult person to express their wishes related to end-of-life care concerning conditions of continuing, of limiting, of stopping or of refusing treatment or medical acts in case they were no longer capable of expressing their wishes. They may be amended or revoked at any moment and by any means.
With the 2016 amendment (Claeys-Leonetti act), these directives acquired a **constraining value** for the medical team and relatives, while previously they only had an informative value; furthermore, they now remain indefinitely valid.
The appointment of a **healthcare agent** is also provided for in article 8, to be a trustee of the patient's wishes in case the latter is not capable of expressing their own wishes. Their testimony prevails over all others. For more detail see annex 3.

In practice, both of these facilities **help physicians**, when the time comes, **to make their decisions** concerning which treatment to give, if the patient cannot express their wishes.
The HAS (French higher health authority) specifically underlines the importance of **prior discussion with relatives** which enables the facility, especially to **prevent their guilt and doubt** when they are faced with choosing for the other person at a difficult moment.

In 2009, only 2.5% of the people who died that year had drawn up end-of-life advance directives, and 6% of them had appointed a healthcare agent according to a report by the Observatoire national de la fin de vie and the IGAS (46). Awareness campaigns have been carried out since then and others are underway, in order to promote this facility.
In Quebec, more than a third of the adult population has signed a "mandate in provision of one's incapacity".

E. Psychiatric Advance Directives

In North America (United States, Canada), in Australia and in several European countries (Switzerland, Netherlands, United Kingdom), **advance directives are tending to spread, covering all situations of decisional incompetence and temporary incapacity of expression**, particularly those of people suffering from mental disorders. (31).

They are only legally binding in the United States, Switzerland and Australia, without legally forcing the physicians to provide the requested treatment if it does not correspond to normal practice.

The aim of Psychiatric Advance Directives (PAD) is to enable a patient suffering from chronic and severe psychiatric disorders **to express their wishes in advance concerning future treatment**, if they were to suffer from another episode of decompensation and find themselves incapable of giving their consent. Advance directives are in the form of a written document, filled in outside the crisis period, while the patient is capable of appreciation and discernment.

They give the possibility of **providing information** concerning the type of mental disorder, the appointment of a healthcare agent, the names of people to contact and not to contact, current treatment, medical history, treatment acceptance or refusal, forewarning signs of a psychotic crisis, efficient treatment during the psychotic crisis and finally provisions of social matter (47) (48)

One of the points that is frequently brought up in literature and in the different information brochures on psychiatric advance directives is when is the best time to write such a document.
The **most appropriate time from a clinical point of view** is the moment **just after the acute phase of the crisis**, when the patient recovers their discernment and is capable of clinically viewing what happened (30,49,50) (51).

1. Different forms of Psychiatric Advance Directives

There are **several forms** of psychiatric advance directives (52).

1.1 "Psychiatric advance directives"

"Psychiatric advance directives" specify certain aspects of treatment that the person wishes (53). A physician can confirm in writing that the person had full capacity of discernment at the time it was written. When a facilitator can be present, 61% of people write the document, versus 3% with no facilitator(54).

1.2 "Joint Crisis Plan"

A "Crisis plan" (United Kingdom) defines what should be done in a crisis, and is signed by the patient, even though they did not write the plan. When it is written with the patient, it is a type of psychiatric advance directive, a prior agreement between the carers and the patient, signed by both parties. This is called a "Joint Crisis Plan" **(55)**. The JCP is only present in research interventions for the moment, it is not used yet in current practice.
The methodology for a "joint crisis plan" follows several steps:

- 1st step, the "Menu": the patient meets the facilitator who presents the different themes that can be addressed in advance directives.
- 2nd step, "Crisis planning meeting": This is a meeting with one member of the care team, the facilitator and the patient, as well as anybody else that the patient wishes to invite. The facilitator encourages everybody to talk.

The facilitator's role is to help the different parties reach a consensus, a "prior agreement". If no agreement is reached, or if the carer did not participate in its elaboration, then the document is not considered to be a prior agreement(55).

1.3 "Ulysses directive[6] ", or "Ulysses contract"

These are advance directives frequently found in the United States and Canada, specifically concerning whether hospital admission and/or specific treatment are accepted or not during potential relapse (48).
For the Ulysses clause to be legally binding, it must be signed either by two physicians, or by a physician and an approved clinical psychologist, indicating that the person had full physical and mental capacity at the time of signature (57).

1.4 Wellness Recovery Action Plan "WRAP"

The goal of this "self-help" plan developed by a group of users, is to draw up an action plan for personal wellness. This plan contains a crisis plan containing a list of events that can lead to an increase in symptoms, and gives the instructions to follow in a crisis (58). It also names an agent who is in charge of applying the instructions given in the plan (48).
As opposed to treatment plans, WRAP is a document drawn up solely by the patient. It is not a legal document. However, it contains more details than the other forms of advance directives (52).

2. Psychiatric Advance Directive Results

2.1 Quantitative PAD results

Quantitative trials on PAD have mainly studied their efficiency to reduce the frequency of hospital admissions and especially admission without consent(48).
For now there is little literature on the subject and the results of these trials are mixed. Randomized controlled trials suggested that they led to a reduction in involuntary admission:

- Use of a "joint crisis plan" gave a significant reduction at 12 months, with 13% of involuntary admission in the experimental group versus 27% in the control group (59)
- a tendency towards reduced admission rates for the WRAP(58)
- Use of a "PAD" led to a non significant reduction of cumulated rates of coercive measures at 24 months (18.8% in the group with supported advance directives versus 27.3% in the other group)(60).

Whereas other trials did not find the same results:

- no significant difference between the experimental and the control groups concerning the rate of involuntary hospital readmission (19% versus 21%)(61). However, in this trial few people had actually filled out the document, as there was no facilitator, as shown in a later study.

The authors of the Cochrane Collaboration meta analysis(47) on advance directives underlined that "*the most intensive forms of advance directives are promising*" and concluded that "*there is currently not enough data to formulate clinical recommendations*".

[6] They owe their name to Ulysses in the Odyssey (56), who, when warned by the goddess Circe of the danger of the sirens' singing but wanting to hear it for himself, asked his companions to strap him to the boat's mast and not obey him if, enchanted by the sirens' singing, he asked them to free him. Ulysses heard the sirens' song but trusty to his "advance directives" his companions did not obey him when he implored to be untied to go and join the sirens.

2.2 Qualitative PAD results

In a study where the objective was to examine the **perception that carers had** of the place of PAD, the authors noted a **valorization of autonomization** of patients concerning their treatment. This study showed that the forms of PAD written in **cooperation with the carer** (Joint Crisis Plan and supported PAD) were more susceptible of being followed than the forms of advance directives written entirely autonomously by the patient.
Indeed, one of the major preoccupations of carers is that the contents of the directives could be at conflict with care practices(62). A more collaborative approach would be susceptible of increasing the person's adherence via the support perceived for their autonomy (63).
For certain authors, writing these directives should be considered "*like **sharing expertise**: the practitioner bringing their medical and scientific knowledge, the patient bringing their knowledge of the disease and their experience of decompensation"* (48). PAD could thus be considered as a support for therapeutic alliance and would make it possible to place the patient at the heart of treatment(64). The perspective is to acknowledge that the person has valuable knowledge about their own problems, that the carer must take into account (65).

Other studies suggested that the implementation of these directives enabled **to reduce the perception of coercion** connected with treatment by patients (66), linked to the fact that "they had their word to say" in decisions concerning their treatment(67).

PAD can also serve as therapeutic tools via **deeper knowledge of oneself and of one's disease** (48) : indeed, several studies have pointed out that people often use PAD to detail the prodromal syndromes and symptomatology during periods of crisis(54,55,68). Working on setting up this sort of document could identify and help the patient identify the forewarning signs of a relapse on the one hand to start treatment as early as possible, and on the other hand to **help verbalization** concerning the illness and its consequences on the patient's life(48).

Widdershoven & Berghmans showed a development of the user's capacity to have a certain **control over therapy**, enrolling them in a **process of appropriation of their problems** (empowerment), and finally the modification of one's own care pathway (69).

So PAD could be considered like an **advance therapeutic teaching, and early prevention of relapse tool** (70) by involving the patient in the analysis of previous crises and in the elaboration of adequate alternative approaches in the future.

F. Intervention in France

Given the potential impact of Psychiatric Advance Directives in France from medical, ethical and legal points of view, **a thesis project** was carried out in 2015 (71), offering 6 patients suffering from psychotic disorders to write their Psychiatric Advance Directives. The psychiatrist in charge of the study first drew up a document addressing the main questions brought up in the foreign protocols. The study quickly showed **the interest of having a facilitator who did not belong to the healthcare profession**, given the time needed for reflection and writing, and the difficulty for patients who had experienced coercion to freely express themselves in front of a classic practitioner. Assessment was performed with semi-structured interviews of users and their referring psychiatrists. The main results obtained were the high level of interest for this approach, and the satisfaction, on behalf of both patients and practitioners.

It just so happened that the facilitator belonged to an organization involved with the question of peer-support for mental disorders: the organization **Eutopia**, and was participating in a research project on the rights of psychiatric patients (project CAPDROIT – organization CONTRAST). Through this facilitator, several members of Eutopia got hold of the PAD tool, used it for themselves and for other people they knew who it concerned.

Thanks to this step of use and coordination, the contents of the PAD writing protocol evolved, and went towards supported writing and sharing via **a peer-worker facilitator**, preferentially taking place "outside the institute". Now, the plan is for PAD writing to become part of a training course at an innovative center: the Centre de Formation au Rétablissement (CoFoR; Recovery training center). The peer-workers working as facilitators for PAD research could be trained together, so that the intervention is uniform and controlled (assessed using parameters defined from discussions between the actors belonging to the project).
A**ppropriation of the tool by users** and the **participatory dynamics** with the integration of members of the organization into the research team, is the first positive result in France.

The interventional trial "Directives Anticipées incitatives en Psychiatrie" (DaiP; Psychiatric advance directives incentive) led by the EA3279 directed by Dr A. Tinland and Pr P. Auquier offers to assess in the most rigorous manner possible the PAD intervention. Right from the beginning this project strongly **mobilized French sponsors belonging to the recovery network**:

- Peer-workers (P. Maugiron, AFMSP - Paris; N. Ordener and C. Letailleur, Eutopia organization - Marseille)
- Physicians: Pr C. Lançon and Pr J. Naudin (APHM), Pr N. Franck (SUR - Lyon)
- Research scientists: T. Greacen (research laboratory EPS Maison Blanche – Paris);

who will host the peer-workers as well as the scientists in their teams and institutes, the research sites;

as well as the organization **CONTRAST** which includes research scientists from several fields: sociology, law, philosophy of the manner in which care practices are regulated, coercion, making decisions for others in the case of people with a disability/ vulnerable/ incapable of giving their consent.

This group of actors anchors the project on both research and intervention.

We formulate the hypothesis that the implementation of Psychiatric Advance Directives facilitated by peer-workers for people suffering from severe psychiatric disorders will reduce at short-term the number of involuntary hospital admissions in the care pathway of these people, compared to individuals who have not benefited from this program.

II. Objectives

A. *Quantitative Objectives*

To assess the impact of Psychiatric Advance Directives facilitated by peer-workers (pw-PAD) in the framework of psychiatric follow-up, in comparison with usual follow-up only, for people with severe psychiatric disorders: schizophrenia, bipolar-I disorder or schizoaffective disorders, with regards to:

1. Outcomes

Primary outcome is reduced coercion assessed by the rate of involuntary hospital admissions over a period of 12 months.

Secondary outcomes are the improvement: of hospital length of stay (reduced duration of free and involuntary hospital stays); the study of intervention costs and its cost-efficiency rate; improvement of the therapeutic alliance; improvement of mental health indicators: recovery, severity, awareness of disorders; improvement of experienced indicators: perceived capacity to act, quality of life; improvement of program user satisfaction and of carers involved (especially the referring psychiatrist).

2. Structures and processes

The assessment of implemented structures and processes will concern the collection of criteria related to structural and human resources, the organization of the pw-PAD and the hospital system with regards to collaborations set up, as well as to practices developed in the framework of pw-PAD and in the hospitals in response to them.

B. *Qualitative Objectives*

The qualitative approach will study people's courses of life, especially their relationship to care and to their rights; institutional dynamics set up around the pw-PAD; studying the representation of users, carers and helpers; and also the conditions making it possible to propagate and spread the program. Particular attention will be paid to legal aspects and ethical aspects.

C. *Participatory Research Objectives*

Being both a research tool and subject, participation represents a crucial element in this research trial.

A participatory approach implies elaboration via collaboration between patients, healthcare professionals and research scientists. This will be present throughout the implementation of the program and will encourage it to proceed correctly.

The primary outcome of a participatory approach is to result in a pw-PAD program integrated both by current clinical practice and by the patients in the experience of the disease and its treatments.

This outcome branches into three secondary outcomes:

- First, to guarantee the effective participation of the patients in the experimental arm in the research program that concerns them, i.e. that patients who wish to can have an actual place in research, that their point of view on the program is heard, understood, valued. The program aims to thus give patients back their voice, give them back their power to act, not only on a personal scale, by drafting their own PAD, but also with regards to the whole pw-PAD program, giving their opinions, reflections or propositions. This effective participation

will be supported by the research team, but also by peer-workers, to explain, inform, listen to, reassure and motivate them. It should propose different research spaces adapted to the people's personality and choice, among the different approaches set up (photography, theater, oral expression).

- Second, to cooperatively improve the organization around the use of advance directives. By opening spaces for speech and discussion between patients, helpers and professionals (peer-workers, psychiatrists, emergency staff), these actors will be led to speak to each other about the issue of advance directives, at several times about their appropriation of the project, to manage to formulate operational recommendations concerning their use: privileged circuit, terms of information, terms of admission, limits... The participation of the different actors in the program's implementation therefore contributes to defining the conditions of the program's implementation.
- Third, carrying out a community sharing of the collected elements. Discussions between patients, healthcare professionals and the research team will be shared with the wider group of actors. This is how to create the conditions to spread the program.

These outcomes are reached through the opening of spaces for discussion between patients, helpers and professionals (peer-workers, psychiatrists, emergency staff), and between these actors and research scientists, allowing a better understanding of each other's issues, with discussions going as from the production/confrontation of hypotheses to a better knowledge of the intervention while including the production of cross-data for the general public.

Different methodological approaches are proposed to do this:

- Peer-workers: they will accompany patients and healthcare professionals setting up the PAD, and will be the privileged witnesses of their implementation, both in their drafting and their application,
- Focus groups: throughout the program work groups will be organized to improve the implementation of PAD and to collect the patients', healthcare professionals' and 'peer-workers' accounts of their experiences,
- Photo-voice: by creating a space for testimonies, photo-voice will add participants' experiences to the program,
- Theater forum: in this collaborative research method, all of the participants will be able to act on PAD implementation.

In all, the participatory approach will be applied throughout the entire program to permit the integration of its implementation.

III. Project steering and partners

This is a multicenter multidisciplinary trial combining hospital and university structures, as well as associative structures.

- Marseille : Assistance Publique Hôpitaux de Marseille, Université Aix-Marseille (EA3279), Collectif Eutopia, CoFoR (Centre de Formation au Rétablissement) ; Centre Hospitalier de Valvert ; Centre Hospitalier d'Edouard Toulouse.
- Lyon : Hôpital Le Vinatier, Service Universitaire de Réhabilitation Psycho-sociale, association Logeurs d'esprit ; Centre hospitalier Saint Jean de Dieu.
- Paris : EPS Maison-Blanche, Laboratoire de recherche de l'EPS Maison-Blanche (GHU Paris), AFMSP (Association Francophone des Médiateurs de Santé Pairs) ; Centre Hospitalier d'Argenteuil – Val d'Oise
- Paris, Lyon : Collectif Contrast (CMW, UMR5283 ; Cersa, UMR7106), brings together research scientists in law, sociology, philosophy who study the recomposition of coercive practices in care, and the different forms that collecting consent can take.

D. Steering committee and scientific committee for the program

1. Steering committee for experimentation

A steering committee is set up. Its mission is namely to note down the indicators monitoring experimentation.

2. Scientific committee

The scientific committee of the "DaiP" program will give its opinion on the composition of the research team. It will validate the different protocols required to reach the objectives fixed for the assessment of "DaiP" experimentation. It will also play a consultative role and offer support to the coordinators of assessment research and to the assessment steering committee. It will give its general opinion on the progress of the trial(s) led in this framework. It can help make difficult decisions for which an independent appreciation is advisable. For example, it can give its opinion in the following circumstances: a premature stop of the trial (or of several), substantial modifications of the protocol, ethical aspects, interpretation of results or requests for further analysis.

All substantial modifications of the protocol must be submitted to it for its opinion. In cases where the objectives fixed for assessment research by the steering committee for experimentation were particularly modified, they should also be validated by the latter. Its composition is the following:

- Pr. Christiane Passerieux, psychiatrist, Responsable scientifique du Centre de Preuves en psychiatrie et santé mentale CH de Versailles
- Mr. Benoît Eyraud, sociologist, collectif CONTRAST and Université Lyon II / Centre Max Weber
- Mr. Olivier Renaudie, doctor in public law, collectif CONTRAST et Cersa
- Mrs. Stéphanie Wooley, administrator of Mental Health Europe, deputy administrator of ENUSP (European Network of (Ex)-Users of Psychiatry)
- Pr. Pierre Le Coz, philosopher, Espace Ethique Méditerranéen, Aix Marseille Université
- Pr. Eric Latimer, economist, Mc Gill University, Montréal, Canada

E. Assessment team

The running of the project is placed under the responsibility of the research team EA3279 directed by P. Auquier, president of the assessment steering committee. The assessment team is articulated around two facilities: an assessment steering committee relying on scientific experts who can be called upon when needed plus a users' committee.

1. Staff

The research team is multidisciplinary made up of:
- a public health physician;
- a psychiatrist;
- a social sciences research scientist in charge of the qualitative section and of the participatory section;
- an economics research scientist;
- a statistician;
- three interviewers (one per site) in charge of collecting data for the "quantitative" section.

The research team, apart form the interviewers who will be present on each site, will be based in the premises of Pr. Pascal Auquier's research unit (EA 3279) at the Faculty of Medicine in Marseille. The team will meet weekly to ensure research monitoring and coordination and will be in charge of the link between the different actors.

2. Assessment steering committee

Composition: The assessment steering committee is placed near P. Auquier's research unit and he will be its president (AP-HM & EA3279 AMU). It is made up of different research scientists directly involved with the research carried out in relation with the experimentation.
Its composition is shown below.

Name	Discipline	Structure
Pr. P. Auquier	Public Health	AP-HM & EA3279 AMU *Coordination team*
Dr A. Tinland	Public Health	AP-HM & EA3279 AMU *Coordination team*
Mrs T. Tartour	Sociology	Collectif Contrast & CSO Sciences Po *Coordination team*
Dr. M. Pontier	Psychiatry	AP-HM *Coordination team*
Mrs S. Loubière	Economics	AP-HM & EA3279 AMU *Coordination team*
Mr. J. Grard	Social anthropology	AP-HM & EA3279 AMU *Coordination team*
Mrs C. Letailleur	Research Participatory Action	Solidarité Réhabilitation & EA3279 AMU *Coordination team*
Pr. C. Lançon	Psychiatry	AP-HM & EA3279 AMU
Pr. J. Naudin	Psychiatry	AP-HM & EA3279 AMU
Mrs M. Coldefy	Health geography	IRDES
Pr T. Apostolidis	Psychology	EA 849 AMU
Dr K. Baumstarck	Public Health Methodology	Unité d'Aide Méthodologique Recherche Clinique, DRCI AP-HM

Missions: Steering committee (SC) missions are the following:
- to oversee the setting up and correct execution of research;
- to oversee compliance with regulations in force related to the research activities;
- to validate the different procedures drawn up concerning the flow of information and data management;
- to validate the definitive versions of the case report forms;
- to validate the procedures necessary to ensure good quality data collection;
- to validate study reports;
- to look for practical solutions for methodological and practical difficulties encountered during research progress;
- to decide about the ways to diffuse research documents and information;
- to validate and oversee the application of operating rules concerning scientific publications;
- to assess whether the goals have been reached, namely in terms of inclusion and follow-up of subjects;
- to decide on potential budget progression, namely by looking for supplementary funding.

For all of the decisions, a consensus of all of the SC members should be sought; in case of disagreement, a decision is made with the majority of members present. The SC meets twice a year. Exceptional meetings may be held at the SC President's request.

3. Users' committee

Expert users selected in the framework of this research will be peer-workers. They will be able to suggest propositions concerning research orientation and will be consulted for all ethical issues. The users' committee will be made up of:

- Philippe Maugiron (Peer-worker Hôpital St Anne – Paris ; President of the AFMSP Association Francophone des Médiateurs de Santé Pairs)
- Allison Symonds (Peer-worker – Paris ; administrator of the association JUST)
- Yves Bancelin (Peer-worker – Service de réhabilitation psychosociale – APHM Marseille)
- Nicolas Ordener (Specialized educator and peer-worker APHM; member of the organization Eutopia, Marseille)
- Anne Bouyer (Participant facilitator at CoFoR, module « vivre avec » - Marseille)

F. "DaiP" project-bearing teams

The teams at the DaiP project-bearing sites are healthcare institutions with recovery-oriented practices in mental health, working in close partnership with the research teams. They also work in partnership with users' organizations.
The composition of the DaiP teams is detailed in Annex 4.

1. Lyon

SUR Service Universitaire de Réhabilitation psycho-sociale (University service of psycho-social rehabilitation) of Pr. Nicolas Franck is a cutting-edge service in France for rehabilitation therapy, and they develop important research on this subject. This structure wants to receive peer-workers and should soon be welcoming a member of the future promotion trained at the CCOMS.
The Hospital Center le Vinatier it is connected to covers 46% of the population of Lyon.
The Hospital Center Saint Jean de Dieu covers three adult psychiatry sectors and has almost 530 beds.

2. Marseille

The APHM psychiatric pole covers six sectors.
The services of Pr Lançon and Naudin already have 6 healthcare peer-workers. They have very close links with the EA3279 in university-hospital missions of the CHU. The EA3279 research team is strongly involved in interventional research in psychiatry and created a status of peer research scientist.
There is an agreement between the APHM and the Centre de Formation au Rétablissement (Recovery training center) (CoFoR) borne by the organization Solidarité Réhabilitation; as well as a partnership with the organization Eutopia about the question of rights and advance directives.
These actors with experience (team MARSS- APHM, Centre de Formation au Rétablissement and organization Eutopia) are ready to support the peer-workers in their role and will be responsible for their training concerning the procurement of advance directives.

The Hospital Center Edouard Toulouse is in the northern quarters of Marseille and covers six adult psychiatry sectors. Psychosocial rehabilitation actions are on the rise.

The Hospital Center Valvert covers four adult psychiatry sectors. The institute wishes to privilege dialog and an open door concerning distrust and confinement.

3. Paris

The Public Health Institute Maison Blanche ensures mental health care for northern and eastern Paris, sectors where social and health issues, associated with the modern day urban environment, manifest themselves clearly. The mission of EPS Maison Blanche cannot only be limited to care. Research and diffusion of knowledge are an integral part of the institute's development strategy.
The research laboratory at the CH Maison Blanche is one of the first research centers in France to have studied recovery and empowerment dynamics, of which Tim Greacen is a recognized specialist. Resources will be mobilized via the Centre d'Empowerment en Santé Mentale which includes peer-workers working in other institutes, such as Philippe Maugiron, president of the Association Francophone des Médiateurs de Santé Pair (AFMSP) who works as a peer-helper at St Anne hospital.

The Hospital Center Argenteuil, in the Val-d'Oise which is the northern agglomeration in Paris, has three adult psychiatry sectors. It is currently being reorganized to privilege psychosocial rehabilitation.

IV. Care strategies under study

A. Pw-PAD intervention

1. Description of the contents and procedure

A person included in the pw-PAD group is incited to fill in the DaiP document "Psychiatric Advance Directives", of which they are immediately given a copy. This document is provided in Annex 5. The person is incited to meet a peer-worker to help them to write it. A peer-worker is called immediately to quickly arrange a meeting, at a place the person has chosen, preferentially in town, outside the hospital.
The person can decide to fill out the document without the help offered.
The person is encouraged to share the filled-in document: with their healthcare agent, as well as with their referring psychiatrist, study investigator, and to give them a copy. A consultation concerning the sharing of this document will be proposed and organized by the peer-worker.
In case of psychiatric hospital admission, the patient hands the document "Psychiatric Advance Directives" to the hospital team, their healthcare agent or referring psychiatrist, so as to influence the therapy given.
PAD incite to follow the preference and choices of the people in question, but the carers are not legally bound to provide the care requested if it does not correspond to practice standards.
PAD do not have any binding value for relatives and carers.

Following inclusion, if the person chooses not to benefit from the intervention, they will be recontacted by the interviewer by phone, taking care not to be too insistent so as not to compromise any future participation. The reminder can be given by the psychiatrist at their next consultation, where the document will be given again, and the peer-worker will again be contacted if the person would like help filling it in.

2. Description of the frequency

The number and the frequency of the meetings with the peer-worker facilitator necessary to fill in the document "Psychiatric Advance Directives" will comply as much as possible with the person's choices and the capacities.

B. *Usual care*

1. **Description of contents, frequency and process**

The contents, frequency and process of consultations with the psychiatrist remain the same.

V. Study population

To be included in this trial patients must meet the following criteria:

A. *Inclusion criteria*

- aged over 18;
- presenting a diagnosis of schizophrenia, bipolar-I disorder or schizoaffective disorders according to DSM-IV classification;
- benefiting from ambulatory follow-up by a referring psychiatrist, investigator;
- having had involuntary hospital admission at least once in the year prior to inclusion;
- having decision-making capacity, assessed by the psychiatrist using a tool inspired by the MacCAT-CR. The MacArthur Competence Assessment Tool for Clinical Research (MacCAT-CR) is a short instrument developed to help clinicians "form an opinion" in the assessment of their patient's abilities. 4 components of decisional capacity are assessed: understanding, reasoning, appreciation and choice. The MacCAT-CR has shown excellent psychometric properties;
- being capable of reading and writing in French making it easier to fill in self-administered questionnaires, enabling a good understanding of questions, enabling discussions with the peer-worker and the research scientist and to give informed consent;
- being able to identify a measure of ambulatory coercion;
- possibility of being under curatorship[7];
- affiliated to a social protection scheme;
- accepting to participate in the trial and with informed consent signed by the individual, as well as information given to the legal curator in the case of a person under legal curatorship.

B. *Exclusion criteria*

- aged under 18;
- participating in another simultaneous trial in accordance with article L1121-12 of the code de la santé publique (French public health regulations);
- being incompetent for decision-making, defined by the psychiatrist using the MacCAT-CR;
- not being able to read or write;
- not affiliated to a social protection scheme;
- refusing to participate in the trial, or the legal curator not being informed in the case of a person under legal curatorship.

C. *Trial exit criteria*

People who withdraw their consent during the trial are withdrawn from the trial.

[7] People under a measure of guardianship are excluded, but those under a measure of curatorship are purposely maintained within the scope of the trial, for representativeness. Inclusion of these individuals complies with legislation. Indeed the Code Santé Publique (French public health regulations) states in article L. 1121-8 that adult persons under a measure of legal protection or incapable of expressing their consent may only be included for category 2 research if research of comparable efficiency cannot be carried out on another category of the population and with the following conditions: (1) either the importance of the expected benefit for these persons is such that it justifies the predictable risk incurred; (2) or this research is justified with regards to the expected benefit for other people in the same situation. In this case the predictable risks and the constraint of this research must be of minimal extent.

VI. Methodology

A. *Quantitative part of research*

1. Experimental design diagram

The trial is multicenter, prospective, experimental, comparative and randomized. It will have two groups of subjects:

- benefiting from the PAD program (Experimental Intervention);
- benefiting from "usual care".

Strategies are detailed in § IV. A. and IV. B. Persons who met inclusion criteria (see § V.A.) are randomly split into two groups from a list drawn up prior to the start of inclusion.

2. Judgment criteria and collected variables

2.1 Primary judgment criterion

The primary judgment criterion is recourse to coercion, assessed by the rate of involuntary hospital admission over a period of 12 months. These data will be collected by asking the patient and the referring psychiatrist and crossing this with data from the PMSI.

2.2 Secondary judgment criteria

Socio-demographic (age, gender, identity, nationality) and social (work situation, income) characteristics will be collected.

Secondary judgment criteria are defined below. There are criteria for assessment of results, institutional and procedural.

a. Results criteria

<u>Criteria related to health</u>

- Anamnestic data and personal medical history;

- Data related to recourse to care and economical data (given in detail in the chapter Economic analysis § D. 3.4)
  - number, duration and type of somatic, psychiatric and addictology hospital stays,
  - number of visits to the emergency department,
  - number of medical consultations,
  - number of sick leaves and reduction of professional activity

- Data related to the therapeutic alliance between the patient and their psychiatrist:
  - Assessed using the questionnaire 4-Point Alliance Self Report (4-PAS), French scale validated showing good psychometric properties with alpha equal to 0.91 (Misdrahi, 2009)(78).

- Data related to mental health assessed from:

  - Recovery, assessed using the self-administered questionnaire Recovery Assessment Scale (RAS) (76,77). Recovery is defined as living a satisfying life despite constraint linked to the mental disease, by analogy with recovery from physical disability, where the person can overcome impairments due to their physical illness and accomplish most of their life goals if they receive adequate assistance and makes the necessary arrangements(76). This scale, validated in a population suffering from severe mental

disorders, demonstrated satisfying psychometric properties(77). It is made up of 24 items, exploring 5 dimensions: self-confidence, capacity to ask for help, life goals, confidence in others, no domination by symptoms. Answers are rated on a Likert scale with 5 points. A global score is obtained as well as sub-scores for the 5 dimensions. Recovery will be assessed using the Recovery Assessment Scale (RAS) at M0, M6 and M12. It has been validated in French.

- 2 Clinical Global Impression (CGI) Scales for the psychiatrist to assess the severity of psychiatric disorders, and their improvement; these scales were developed in the United States for research on schizophrenia. They have not been validated strictly speaking, but they are widely used nowadays in the majority of clinical trials(74). The 1st CGI concerns the severity of the illness. It is rated from 0 to 7, with 1 being the normal state, 0 not assessed. The physician is asked to grade the patients according to his/her general clinical experience of this type of patient, whatever the current level of severity of the patient's mental disorders. The 2nd CGI concerns global improvement. The physician is asked to assess the global improvement of the patient compared to their state upon trial inclusion. This CGI also has 7 levels.

- Awareness of one's own disorders, or insight, measured by the SUMS (Scale to Assess Unawareness in Mental Disorder)(79) which is one of the most widespread instruments for assessing this parameter, and which demonstrated good psychometric properties. As did the scale in French, where a study confirmed the 3-dimensional structure: awareness of disease, of its disorders and of the need for treatment / awareness of positive symptoms / awareness of negative symptoms; it showed good reliability (alpha 0.85) (Michel, 2013)(80). The original version with 74 items was shortened to improve acceptability and thus make it easier to use in clinical practice. In this trial we use a version with 9 items that is filled in by the clinician after a structured interview with the patient. The following 9 points are considered, awareness of: a mental disorder; consequences of this disorder; effects of drugs; hallucinations; delusional ideas; troubled thoughts; blunted affect; anhedonia; lack of sociability. Each item is graded from 0 to 3.

- Data related to the patient's experience assessed using:

  - The Empowerment Scale (ES) has 28 items concerning 5 dimensions: self esteem, power, activity, fair anger, optimism. This instrument is widely used and has been translated into several languages, including Portuguese and Japanese, without modifying its 5 factor structure, and keeping psychometric characteristics. It was translated into French in Quebec (context bilingual Canadian), and is already used and available in this form. However it has never been validated in actual French.

  - Health-related quality of life will be measured using a specific scale: the S-QOL. This is a self-reported instrument of 41 items which assesses the quality of life of patients suffering from schizophrenia using 8 sub-scales: psychological well-being, self-esteem, relationship with family, relationship with friends, resilience, physical well-being, autonomy, love life. This is a validated French scale(82). The short version containing 18 items demonstrated excellent psychometric properties and will be used in the trial (80). The instrument was validated for the bipolar population (83).

- Data related to satisfaction, assessed using:
  - Client Satisfaction Questionnaire-8 (CSQ-8) which is the satisfaction questionnaire the most used for the assessment of mental health services. It has been translated into over 20 languages, including French. Originally developed as an 18-item version (CSQ-18) with very good internal consistency: alpha = 0.91 (84), this one-dimensional tool has been validated as an 8-question version. Each question is graded form 1 to 4 (4 point Likert

scale), scores can vary from 8 to 32. High scores indicate satisfaction, which is what is expected in this program.

- Data related to people lost to follow-up.

b. Institutional and procedural criteria

Indicators of institutions and procedures will be systematically collected and will concern human resources, institutional resources, the actual number of directives filled in, the number of people who had recourse to a peer-worker, the number of contacts with the peer-worker required to fill in the document, the number of shared directives and with which actor(s), use of the document in case of hospitalization, anticipated directives respected or not in the case of hospitalization, the number and type of collaborative link with local partners, description of the actions performed by the peer-worker, etc. Identification of future dysfunctions, their origins and the corrective measures applied.

3. Data collection periods

Individual case report forms have been developed especially for this trial; they are articulated around the three assessment periods described in the following chapter: initial assessment and follow-up visits at 6 and 12 months.
Data will be collected at a place of the patient's choice.
Data will be manually entered into a laptop dedicated to this use, using software specifically developed for the trial.

The following table summarizes the contents and the frequency of assessments for the two groups of subjects included in the trial.

	Eligibility Inclusion Init. Ass.	6 months	12 months
Informed consent, check inclusion/exclusion criteria: admissibility questionnaire	x		
Sociodemographic characteristics	x		
Anamnestic data & personal medical history	x		
Data related to mental health: CGI	x		x
Recovery: RAS	x	x	x
Therapeutic alliance: 4-PAS	x	x	x
Insight: SUMS	x	x	x
Empowerment: ES	x	x	x
Quality of life: S-QOL	x	x	x
Satisfaction CSQ-8	x	x	x
Data related to recourse to care system	x	x	x
Data related to indicators of institution and processes, lost to follow-up	x	x	x

B. Procedure

	When?	Who?	Interviewers Y/N
1. **Detection – Eligibility**<br><br>The psychiatrist investigators in charge of detecting subjects in their active file of patients will be given precise information about the trial before the recruitment period.<br>These psychiatrists are in charge of checking that the patient does meet the psychiatric inclusion criteria for the trial and of giving the type of diagnosis: schizophrenia, bipolar-I disorder, schizoaffective disorders.<br>During a meeting with a potentially eligible patient, the psychiatrist fills out the eligibility questionnaire, then once all of the eligibility criteria have been validated he/she contacts the research team investigator and faxes the filled out form serving as a "request for inclusion".<br>The psychiatrist is in charge of giving the patient information about the "DaiP" intervention and its objectives. He/she hands the patient the **information sheet** and then goes over the assessment goals for the "DaiP" intervention, the procedure, the advantages and the constraints.<br>An appointment is made in the next few days for a meeting between the psychiatrist investigator, the patient and the interviewer.	D0	Psychiatrist investigators	N
2. **Inclusion**<br><br>The subject is recruited during this meeting.<br>If the patient is under legal protection, the legal representative is informed.<br><br>The interviewer and the psychiatrist make sure that the person has correctly understood and obtain his/her **informed consent,** or that of the legal representative where appropriate, after agreement by the patient.<br>The subject can be allowed a reflection period of one month maximum before giving consent to participate in the trial and in this case a second appointment is made.<br><br>Next patients are randomized. The patient is informed of their inclusion in the "PAD" or "Usual Care" arm of the trial.<br><br>• If the person is included in the "PAD" arm, the physician investigator immediately offers a meeting with the peer-worker. The interviewer makes the next appointment and takes the person's contact details. The referring psychiatrist, investigator, is in charge of informing the person's family doctor and the actors involved in care that the person will be participating in this research trial. This information is sent by post, and may enclose the sheet given in Annex 6.<br>• If the person is included in the "usual care" arm, the interviewer makes the next appointment and takes the person's contact details.	D0 to D5		Y

3. **Initial assessment**<br><br>The interviewer fills in the sections of the case report form corresponding to initial assessment.<br>A calendar is defined between the patient and the interviewer to plan the meetings required for the trial. The interviewer must again go over all the possible means of contact (phone if possible) for appointment reminders.<br>The assessment must be done **soon after inclusion** within a maximum of **one month after inclusion** and **before the intervention starts.**	D0 to D30		Y
4. **Follow-up**<br><br>Follow-up visits take place 6 and 12 months **after inclusion** in the trial which formally corresponds to the date the informed consent form was signed. Follow-up assessments will be valid if their dates are within a period of +/- 30 days of the scheduled date.<br>According to the means defined with the patient, the interviewer will contact the patient before the follow-up visit, then go to the scheduled meeting at a place that suits the patient. At each visit the interviewer fills in the corresponding sections of the case report form.	M6<br>M12		Y<br>Y

C. Number of subjects necessary and feasibility

1. Number of subjects necessary

Subjects included in the trial are people who were involuntarily admitted to hospital within the previous 12 months. According to data in French literature, the rate of involuntary hospital admission for patients suffering from psychotic disorders is 42.6% within 12 months after their previous admission(27).
Reducing these involuntary admissions by one third, i.e. 33%, would be a very significant reduction in terms of efficiency. This is a realistic hypothesis in the light of the most recent meta-analyses (59). We consider that 10% of participants will not reach experimental intervention and we estimate a 30% size effect. With these hypotheses, for an alpha risk of 2.5% (one-sided test) and 80% power, 182 subjects per group would be necessary. Over a 12-month period rate of lost to follow-up equal to 10% has been considered acceptable by the investigators involved in this project, especially with regards to the means scheduled to limit the number of people lost from sight.
Accordingly, the total number of subjects to include is 400 patients.

2. Feasibility – Trial duration

2.1. Scaling up

Inclusions will be progressive as the patients present inclusion criteria (open trial), and as the peer-worker and interviewer are able to meet new people.
Recruitment of patients will be progressive, until reaching 150 people per site for the Lyon and Marseille sites, 100 people for the Paris site, over an estimated period of 12 months. For each subject the duration of follow-up is fixed at 12 months. In total, this trial will have a duration of 30 months including 6 months of analysis.

2.2. Recruitment

Estimated inclusion rate is 8 to 9 patients per months for the Paris site, and 12 to 13 patients per month for the Marseille and Lyon sites. These estimates are considered to be realistic regarding the active file of the interviewers involved on the different sites. They have been used as the basis to calculate the workload of peer-workers and were used when drafting the budget for research operation.

2.3. Strategy to limit the number of patients lost to follow-up in the "usual care" group

Several methods are used to limit patients lost to follow-up.

- The investigators are in charge of recruiting patients from their active file. These patients are correctly followed.
- A systematical questionnaire will give several means of contacting the person: landline and cell phone numbers, address, e-mail address, facebook… If the person agrees, the interviewer can also take down contact details of family or friends, in case of loss or change of cell phone, or a change of address for example. This information will be updated at every meeting.
- Finally, the interviewers have the task of creating a relationship based on confidence, knowledge and recognition which will strengthen this link over the months of survey. If necessary, and upon the interviewer's assessment, with the person's consent, appointments will be made for physical informal meetings between survey meetings, to strengthen the link.

In case of non reply, the psychiatrist and the support team will be mobilized.
The interviewers will be careful not to be too insistent, so as not to persecute patients.
Each case will be discussed in the light of ongoing respect for the person.

D. Data processing

1. Data sources

All data are recorded from an electronic case report form (eCRF) specifically elaborated for the study (eCRF CleanWEB, Telemedicine Technologies S.A.S., www.tentelemed.com, 2015) and recorded at four specific study's times as follows: baseline assessment (T1), at 6 months (T2), and at 12 months after inclusion (T3). All assessments are based either on medical database and face-to-face questionnaires

2. Data quality control

The e-CRF will be set up to allow real-time performance of the main consistency controls.

3. Data entry

All data will be entered into the e-CRF on a secure laptop provided to the interviewer. These data will be entered from data sources kept by the psychiatrist (clinical data, assessments, scores…). Consistency controls will be carried out in real-time. Questionnaires will be filled in directly with the patient.

E. Statistical Plan Analysis (SAP)

This plan was prepared by Anderson Loundou and Sandrine Loubiere and validated with the research team on 15 January 2019. There were no changes in this SAP during the study.

1. Analysis Populations

The inclusion criteria are as follows: being over 18 years of age; having a diagnosis of schizophrenia or bipolar I disorder or schizoaffective disorders according to Diagnostic and Statistical Manual of Mental Disorders, fifth edition (DSM-V) criteria; having been compulsory hospitalized within the past 12 months; having decision-making capacity assessed by psychiatrist in the four component abilities of a decisional capacity standard: understanding, appreciation, reasoning, and choice, from the MacArthur Competence Assessment Tool for Clinical Research (MacCAT-CR); being covered by French government health insurance; and speaking French. Exclusion criteria include the following: being considered unable to provide informed consent and being under guardianship.

2. Effectiveness Analysis

2.1. Primary Outcome Measures

The analyses of the primary and secondary criteria will be performed on the intent-to-treat population. In addition, complementary per protocol analyses will be performed (that is comparison of persons who completed the intervention originally allocated).

The primary data will be summarized using frequencies. Comparisons between the two groups will be performed using chi-square or Fisher's exact tests for proportions. Multivariate analyses will be performed for the rate of admission using logistic regression. No adjustment will be carried out in the primary analysis.

Statistical analyses will be performed using SPSS Statistics for Windows, Version 20.0 (SPSS Inc. Chicago, IL, USA) or STATA 16 (StataCorp. 2019. Stata Statistical Software: Release 16. College Station, TX: StataCorp) LLC.

2.2. Secondary Outcome Measures

The data will be summarized using the mean, median, standard deviation and range of quantitative data. Mean score values will be compared with the Student t test or the Mann–Whitney U test. Secondary outcome scores will be analysed using generalized linear models with a normal distribution with an identity or log link.

The cost-utility analysis will be carried out according to the recommendations of the French Health Authorities (HAS). The final result of the economic analysis is the incremental cost-utility ratio (ICUR) that is the ratio of added cost to added effectiveness, which expresses the additional costs per additional QALY. Univariate sensitivity analyses will be carried out by varying health and cost parameters. As recommended by the HAS, the statistical uncertainty surrounding the ICUR will be captured by a probabilistic analysis, using, among others, simulation methods such as non-parametric bootstrap methods.

3. Safety analyses

As this research is part of the research involving the human "at risk and minimal constraints" (category 2 in Jardé Law), and in accordance with the regulations in force, it will be up to the investigator to declare any serious adverse event that occurred during the investigations according to the internal procedure of declaration of a serious adverse event associated with the care of his institution. The sponsor will conduct study quality monitoring. The project can be audited by the French competent authority in case of non-respect of the safety or the rights of the participant.

Adverse event: Any noxious or undesirable event experienced by a participant during the trial, whether or not considered related to the intervention should be considered as an adverse event (AE).

Serious adverse event: A serious adverse event (SAE) has to be reported to the sponsor within the 48 hours of its occurrence. SAE is an adverse event that is: Fatal (even if not related to the disease); Life-threatening; Significantly, persistently or permanently disabling ; Requiring intervention to prevent permanent impairment or damage ; Any event that could be considered as potentially harmful ; Any event medically accurate according to the investigator's judgment.

These serious adverse events will be cautiously reported in the CRFs.

4. Sensitivity Analyses

As a sensitivity analysis, the regressions will be repeated including random effects for sites, and adjusted hazard/odds ratios will be provided based on potential un-balanced baseline characteristics or relevant covariates (such as diagnosis and age). Known or expected associations with the primary outcome variable in our study will be on the basis of previous evidence (e.g. previous publications) and/or on clinical grounds.

Sensitivity analysis will be carried out to address uncertainty around the cost-utility ratio. One-way sensitivity analysis and tornado diagram will be performed to identify thresholds for factors

influencing the ICUR. Probabilistic sensitivity analyses, using the non-parametric bootstrap method, will be carried out to generate mean expected ICURs and to determine whether uncertainty or variation in the data used affect the ICURs. In addition, cost-effectiveness acceptability curves will be drawn to represent decision uncertainty surrounding cost-utility estimates.

5. Other analyses

NA

6. Handling of Missing Data

Missing data will be handled where possible using multiple imputations or other method according to the type of missing data.

7. Interim, Final Analyses and Timing of Analyses

No interim analysis scheduled.

F. Qualitative assessment

The guiding principle of the qualitative section is better theoretical and practical understanding of recovery processes.
Recovery has been conceptualized by American scientists as a singular process that is not only biological but also social, civic and existential (38). For them, *recovery* concerns not only the course of illness but also the course of life and particularly restoring social ties and the person's autonomy. In this line of action, our approach consists in understanding the forms of life and the specific processes that enable a person to hold on while going through life's challenges and mental illness, and this, whatever the manner they are externally defined in. Particular attention will be paid to representations and awareness of rights (87,88) of the different actors involved in the recovery process.
In synergy with the randomized trial, social anthropology research scientists will purposely place themselves in a perspective of triangulation. Triangulation of data enables to add depth to the collected points of view by revealing new facets of the phenomenon under study and trying to take into consideration the *changing dimension of encountered problems*. This triangulation consists of collecting data from program operations and accounting for the examination of social dynamics present within its implementation. The qualitative approach attempts to open breaches, tries to account for blanks in order to reintroduce a better understanding of the processes at stake at different levels of reality.
On the one hand, it is to assess the expected effects of the program on the individual beneficiaries and on the other hand, to assess its expected but also unexpected effects on relational and systemic dynamics. This perspective gap, once spelled out further, justifies the importance of choosing differentiated methodologies the best adapted to the goals.
The presentation below is split into three parts. The first part explains the chosen themes of investigation, as well as why they were chosen. The second part presents methodology retained for these themes. Finally the expectations of this research project are described.

1. Challenge and presentation of the themes of investigation

What can one expect from legally provided tools from a recovery perspective? For research scientists, professionals, users, politicians, the answers may all be different. From a pragmatic social perspective, the different themes presented below will be honed down progressively as the research progresses: whether it is creating a multidisciplinary discussion within the research team, the reflexivity of professional participants, experimentation and the users' opinions and more broadly the evolution of engaged public action instruments.
Interrogations concern:

- Institutional dynamics implemented around the program;
- discussions about the form of advance directives and their possible contents;
- pathways for care and recovery;
- the participatory dimension.

This research program aims to understand how the three categories involved in each of the themes (institutions, professionals and users), while apparently having expectations on different and sometimes opposite horizons, construct tools for regulation, negotiation in order to make the program "hold up" giving it common social sense. Indeed there is inbuilt tension between these themes; action reference frames, goals and challenges are specific to each of them. The documented assessment of how tensions build up and are released within each theme and between the themes will finally be of assistance in choosing policies for the future spreading of the program. In this way, the qualitative section includes a form of assessment research.
After the final report (quantitative and qualitative parts) we propose to translate the research results into recommendations. The method: local monographs and comparative approach.
The program was implemented at several sites, its qualitative assessment will take place at each of them, which will favor a transversal comparative approach and will account more for local contexts. Comparison will be made possible by using the same tools at each site. Then we shall describe how the issue of transversality will be translated, in the inter-site comparison as well as for the triangulation implemented for the epidemiological part of the survey.

2. Methodological tools

On each site, the same methodological tools will be offered, making it possible to question each of the three targeted categories of actors: institutional, field practitioners, helpers and program users. There will be adaptations during the survey in order to adapt the approach to the specificities of each field.

a. Survey of institutional actors: focus groups and interviews

Interviews are scheduled to get to know the local institutional scene and its history: why is experimentation possible there? What vision of the legal instruments of anticipation do the different actors have (professionals in psychiatry, users, militants, helpers)?
Various actors (psychiatric department, organizations) can be interviewed.
Interviews are the preamble to setting up focus groups. The categories of actors in the focus groups will be determined site by site. Focus group topics will be determined with the participants as the actions progress. The first *exploratory focus group*, acts as the first step to allow topics to emerge that seem pertinent for the participants. After these exploratory focus groups, focus groups will be focused on the questions.
The focus group technique implies the presence of three people, one who organizes the debate in a distanced manner, one who plays the role of peer-worker and refocuses the debate, and one who observes all that is non verbal and sharp interventions which would be missed by the audio tape. One of these people will be a user (position to be defined). There will be a debrief afterwards (of at least one hour).
Focus groups will be taped and transcribed. The meetings will be held once per term. The aim is to go back over dynamics of actors, interactions, short, medium and long-term consequences. Focus groups will also be the privileged means of reaching community expectations, shared by the

participants, faced with this new approach, but also their fears.

For example, if we take Marseille, a focus group could include:
- trial participants
- people who had already had PAD before (ex: during the pilot study by Dr Pontier);
- facilitators from the organization Eutopia;
- the peer-worker;

b. Survey of field practitioners: interviews, participatory observations and focus groups

The survey of field practitioners will first be **direct and participatory observation**. This enables to:
- document the conditions of a problematic or promising pathway;
- understand the points of view of the different protagonists;
- propose a descriptive report of coordination and negotiation at the time they take place (cross processes and data with what the actors remember);
- note down consensual or non-consensual logic as it is expressed in action to understand the agreements and disagreements at hand, and vice versa.

Participatory observation requires the definition of "target situations" (interactions between professionals and users, between peer-workers and professionals or users for example, with the consent of the participants) and the frequency of observation.
A regular logbook of the frame of discussion is held throughout the program.
Particular attention will be paid to the scope of problematic and promising situations and the manner in which they are defined.
Interviews are scheduled with professionals as the *program progresses*. They will help to discover:
- the actors' point of view concerning their practices;
  - axiology;
  - praxeology;
  - temporality;
  - resources;
  - obstacles and limits;
  - their assessment of results;
  - evolution of their expectations, etc.

If interviews, and this is where the strength of the complementarity of ethnographic methods lies, are coupled with the direct, enable the measurement of the gaps between speech and practice. It is not about "criticizing" the professionals' practices, but really and truly about confronting what is at play in daily interactions and which, having become mundane, or even trivial in the eyes of the actors, is often missed in interviews. An interview situation is effectively an artificial social situation, during which the actors are disconnected from their usual social context of enunciation related to their practices. Thus the interview gives access to *praxeology* – speech related to practices – while observation enables to study speech put into action, the actors' practices. However, this decontextualization which is the basis of an interview, physical -since the actors are not "in place" – but also cognitive and social, make it possible to not be trapped in daily imponderables – also implying for them the possibility of taking the back seat, which brings added data and sense compared to observation alone. Nevertheless, the interview situation, while it allows access to their analysis of situations, practices and values, is at risk, depending on the context, of only normative and artificial speeches.(89).
This is why putting the data collected during the interviews alongside those collected during the observations will be a crucial bet. This is even more important and primordial since we sometimes process "sensitive" issues. (90).

The survey of professionals will also include setting up **focus groups** (Morgan, 1997) with the different psychiatrists in the program, as well as with professionals from the psychiatric emergency

department. The theme of these groups will be *problematic pathways*. They will go back over those which are controversial or cause tension, in order to re-elaborate categories of analysis, go into more depth about strong points but also about detected blind spots. The objective in a culture of participatory action research will be double: document problematic pathways and think about a better adjustment of practical responses. Focus groups will be organized every term by the research scientist.

c. Survey of users

This is the reconstitution and analysis of the users' **capacity pathways** (91).

This notion takes into account the temporality of civilian life, including both biographic dimensions (physiological, experiential, relational) of life experience, and social regulation by rights, especially in life situations where restrictive, involuntary actions have been lived through. The proposed phases articulate the different issues of legal regulations, like the qualification of facts, putting forward ideal principles, the problem of the applicability of these principles and, finally, that of adapting legal regulations.

Data from 6 individual pathways will be collected per site, i.e. 24 pathways. This will be done on a voluntary basis: if there are more volunteers than desired, we will choose by drawing them from among the candidates proposing their pathways. Thus one person per site per month will be interviewed for the duration of the inclusion period.

Out of these 6 pathways, 4 belonging to the "DaiP" group from each site will be documented. The 2 others will be from the "usual care" group. For the people from both groups, there will be a type of biographic, exploratory interview, in order to collect prior capacity pathways. This first wave of interviews will be used to draw up a series of ideal-typical profiles (92) of people concerned who volunteered to participate in this research. Each person will be interviewed every 6 months over the 12 months of follow-up (i.e. 2 interviews per person).

This method has the advantage of documenting pathways at the different moments of build-up in the program for each of the groups and thus obtain a varied palette of situations.

The term interviews, semi-structured, will be about what the subjects feel, their respective experience of the approach, with the aim to assess a potential evolution of these models, of these ideal-typical figures. Comparing the life experience of people included in the "DaiP" group with the others while experimentation is in progress like this, we should be capable of measuring the subjective effects of the program, *in action*.

A final interview will take place at the end of experimentation, in order to collect, from a participatory perspective, subjective feelings and experiences.

d. Specific facilities for the participatory approach

The issue of the conditions in which users will be speaking, is an imperative in this trial to understand what the program really does from this point of view.

We propose to build *testimony spaces* which open up the possibility of a diversity in points of view, which notably overcomes the obstacles related to formal terms of discussion, to the limited period of experimentation and to the lack of knowing each other before between users presumably unorganized compared to the fixed frame of the program.

The issue of healthcare professionals speech is just as important to accompany and mark out possible changes in practice, to transcribe better what is at stake in the implementation of PAD, and finally to formulate solid propositions to improve-specify the intervention.

Methodologically and in compliment to the interviews and focus groups described above, we propose **spaces for co-elaboration of research** in the **theater forum**; and for **witnessing** using the methodology **Photovoice** (4)**.**

Photovoice is a method of collaborative research enabling a better understanding of a subject and an intervention, and aims to improve the conditions of both via collective action. Photovoice is a project with a collection of informative pictures, and the legends of these pictures, from photos about a subject taken by the members of a group. Photovoice progresses together and step by step, resulting in a display of photos and an analysis grid on the subject by the group formed, in which the participants become co-creators and co-researchers. The participants particularly appreciate this method (93).

The analysis grids by the different participants (patients, professionals) will be useful for posing problems and for communicating. The displays from Photovoice projects make it possible to directly address the general public.

In the framework of this project, the groups formed will be a group of professionals (psychiatrists, emergency staff), and a group of patients.
One training session with a photographer will be organized per group (on the use of cameras, on pictures: perspective, contrast or other techniques used to translate the experience). At the end of the session each participant will be given a camera.
Thanks to this tool, and in 8 sessions led by the research scientist according to Photovoice methodology guidelines, each of these groups will have to answer the following two questions:

- What are the main obstacles for the implementation of PAD?
- What are the outcomes of PAD efficiency?

The reports produced by the groups (selection of photos with titles, topics) will be analyzed by the research scientists, who in turn report back to the groups.
A consensual version of the reports is produced. The photos are definitively chosen and captioned. A restitution-exhibition is organized around the photos, representing session 8.
The participants are given a copy of the photos if they wish.

Theater-forum proposes to use theater play to encourage verbal expression and reflection on a chosen theme. First, actors play short scenes evoking blocked or conflict situations. Then the scenes are acted again with one of the public replacing one of the characters or creating a new one to try and reach a more satisfactory solution. The "spect-Actor" tries out possible alternatives against the difficulties. This is a fun and interactive tool adapted to implement dynamics of change (5).
Scenarios built around PAD and played by both professionals (psychiatrists, emergency staff) and patients could improve the program, its implementation and discover operational recommendations. The progression of scenarios over time will be analyzed as a marker of appropriation of the program. A synergy between testimonies and pathways between 0 and 12 months could help understand why the users are able or not to make citizen spaces of participation their own.
Finally, we emit the hypothesis that PAD are literally empowerment tools, and for this reason they enable to go forward, to place milestones along the pathway to the recovery of health and a social life. They are much more than just a tool to prevent hospital readmission: they enable users to get out of a subaltern or marginal position, and to (re)become a citizen, if not entirely, in any case much more than before.
These testimony spaces built on this participatory methodology, coupled with the interviews, will allow us to measure this dimension.

2.1. Transversality

a. Specificities and comparison between sites

The senior sociologist will hold a transversal position at the different sites, and will go on site periodically. He will not be in a position of proximal observation. He will periodically attend assessment steering committee meetings. He may also be invited by the users' commission if necessary.
The geographical distance between the sites may be a boundary to global consistency, as it limits exchanges and communication. Inter/multi-disciplinarity must be valorized by establishing a frame facilitating discussion. The data from each of these sites will be transcribed and processed by software that sorts and classifies material collected, coded and then analyzed using the same tool (type inVivo).
Due to these risks, it is necessary to establish a stable framework uniting the research team in its entirety. A **team seminar** will be held four times per year. It will be led by the research coordinators (quantitative and qualitative) and will be made up of on-site research scientists and interviewers. This seminar should enable to identify methodological difficulties. It will allow everyone to bring their results and to confront them. This seminar will have the role of homogenizing the methods (interview grids, choice of situations to observe, focus group topics; Photovoice, theater-forum) and data collection from the different sources. It should also stabilize the categories for analysis and thus

make it easier to lead a research policy that is compatible between sites.

b. Triangulation: open seminar for epidemiology research scientists

Across the Atlantic, the approach consisting of associating various methods has been called “triangulation”, a military or marines science term for the joint use of several methods, particularly quantitative and qualitative to give better answers to the questions asked about a given subject. It is necessary to cross views at the different stages of quantitative and qualitative research (grid, answering questions, assessment scale, seminar for data recovery).

3. Summary table

THEMES	Individual and institutional dynamics around advance directives	Persons' care and recovery pathways	Citizenship, participatory dimension
**Objectives**	- document the specificity of the sites<br>- understand partner dynamics internal or external to the paper-program<br>- brakes and facilitators of implementation	- document 24 care pathways on four sites (6 / site)<br>- identify the resources mobilized by the users<br>- document problematic and promising pathways<br>- pay attention to adjustments of temporality	- guarantee participation<br>- document the appropriation of new citizen skills among the users<br>- guarantee the presence of discussion spaces between the program's actors, from healthcare and research
**Research scientist**	0.4 ETP senior sociologist coordinating a one-quarter-time sociologist-interviewer per site for data collection	0.4 ETP senior sociologist coordinating a one-quarter-time sociologist-interviewer per site for data collection	0,1ETP sociologist Photovoice and theater-forum led by the sociologist-interviewers
**Transversal methodology : regular team seminars**	- Focus groups with carers, helpers, users and institutional representatives	- Participatory observation<br>- Interviews<br>- Focus groups	- Construct testimony spaces<br>- Photovoice: exhibitions<br>- Theater-forum: videos
	Transversal seminar / Crossed quali/quanti participation		

4. Expectations: the question of generalizing the program

One of the main objectives of this program, if it proves its efficiency, is to generalize it. From this perspective, the original programming is as much at stake as its implementation on the different sites.
Rather than reproducibility which supposes identical duplication that is actually impossible (the actual experimentation conditions are never identically renewed), we prefer to talk about propagation and spread. So the qualitative question is the following: which conditions would give the generalization process a chance to succeed?
Throughout the assessment research work, the pioneer effect must be accounted for in analysis. One can suppose that the program will generate some adhesion but also some resistance which need to be documented, but also, in return, forms of creative action with some of them being specific of a "pioneer effect", while others will be more common. For each of the themes, we shall document the question of a potential generalization from the observation of particular contexts presented to the actors as experimental. Thus, the choice of implementing the program on four different sites is a real advantage form a methodological point of view. It will favor a distinction between specific and transversal elements.
With this in mind, the aim to describe the program's effects on the health and social organizations

and on the professionals' and users' representations and practices will help to understand the conditions in which a generalization of the program would be possible.

VII. Calendar

The provisional synoptic calendar of the trial is shown below.

Date	Action
M-3	Open the budget line with the managing body
M-3 to M-1	Recruitment of the research team<br>Training of interviewers<br>Training of psychiatrist-investigators<br>Recruitment and training of 4 peer-workers<br>Preparation of the different documents (questionnaires, case report forms, consent forms, information sheets, randomization lists)<br>Test phase for questionnaires<br>Validation of documents by the assessment steering committee<br>Application for authorizations: CPP ethics committee
M0	Obtention authorizations: CPP ethics committee<br>Opening/setting up recruitment centers<br>Start of inclusions
M12	End of inclusions
M24	End of follow-up for last patient<br>End of data collection
M25	Quality control
M25-M30	Data analysis
M30	Validation of results from final analysis<br>Organization of data valorization

VIII. Consistency with regards to research policies, perspectives and feasibility

A. Consistency with regards to health policies

The project perfectly fits in with international (WHO) and national (Stratégie Nationale de Santé, DGS) policies.

A. 1. On an international plan, the World Health Organization (WHO) underlines the importance of protecting the rights of people with mental disorders, who constitute a vulnerable section of society, confronted with stigmatization, discrimination and marginalization in every society, which increases their risks of violation of their human rights (94).

WHO published directives concerning the rights of persons suffering from mental disorders in 1996 (95). Among the 10 fundamental principles related to mental healthcare, 4 are particularly concerned by the DaiP program.

- **Promotion of mental health and prevention of mental disorders;**
- Access to basic mental health care;
- Assessment of mental health in compliance with internationally accepted principles;
- **Provision of the least restrictive type of mental health care;**
- **Self-determination;**
- **Right to be assisted in the exercise of self-determination;**
- Availability of review procedure;
- Automatic periodical review mechanism;
- Qualified decision-maker;
- Respect of the rule of law.

WHO recalls the principle of free and informed consent to treatment, and the exceptional character of involuntary treatments.

It quotes the **principle of the least restrictive solution** (p21) as one of the fundamental provisions in mental health care legislation

> "All people with mental disorders should be provided with treatment based in the community except in very rare circumstances e.g. if there is a risk of self-harm or harm to other people or if the treatment can only be provided in an institutional setting. If institutional admission or treatment is necessary, legislation should encourage this on a voluntary basis."

A.2. In France, health democracy, rights of users and patients are transversal themes in all recent texts for healthcare policy orientation.

- The DAiP project fits two of the three themes of the **loi de santé (French health law)**, promulgated on January 26, 2016: reinforcement of prevention, and the development of patients' rights.

- The **decree relating to territorial mental health projects**, published on July 27, 2017 is the 1st structural text for mental health in the last 20 years. It fixes the 6 priorities which every territorial project must answer:

- early detection of mental disorders, elaboration of a diagnosis, improved access to care and social or medical-social accompaniment
- organization – with no rupture – of people's pathways, in particular those with serious disorders, at risk or in a situation of mental disability, for their recovery and social insertion
- access to somatic care for people suffering from mental disorders
- prevention and care in crisis and emergency situations
- respect and promotion of the rights of people with mental disorders, reinforcement of their power of decision and action as well as fighting against the stigmatization of these disorders
- action on social, environmental and territorial determinants for mental health.

<u>The DaiP project perfectly responds to least 2 of these 6 priorities for territorial mental health projects</u>: 1) respect and promotion of the rights of people with mental disorders, reinforcement of their power of decision and action as well as fighting against the stigmatization of these disorders 2) prevention and care in crisis and emergency situations.

- Theme 2 of the **Stratégie Nationale de Santé (French national health strategy) (2013)** insists on better care to avoid complications and unnecessary hospital admissions; theme 3 is entirely devoted to the reinforcement of health democracy, marking the wish (3.3) to involve and accompany patients and their representatives in the organization, functioning and evolution of our health system.
  It announces new provisions aiming to ensure full participation of patients, their relatives and their representatives, mentioning the importance of reinforcing users' community rights, in line and going further than the **act of March 4, 2002** which consecrated the notion of ill peoples' rights and enabled important progress by setting up a better representation of users, as well as patients having the right to direct access and entirely dispose of their medical records, and finally compensation for therapeutic hazards.

- The act of "supporting" users' health system rights to help reinforce democracy in health" is also at the heart of the **DGS** strategy, which places information and participation at the base of rights, highlights co-construction, and proposes to collectively invest in health promotion and prevention (priority 1); to promote an inter-sector approach; to valorize non-institutional actors in health promotion, whether the population itself, organizations, or all healthcare professionals, for an operational implementation the closest to reality on the field.
  "The DGS strategy must integrate the recognition and the preservation of users' rights, whether it is the patient, actor of his/her own healthcare pathway 'pactient' or his/her representatives".

- The **Conférence Nationale de Santé** recommends the integration of users' organizations.

  - The **Haute Autorité de Santé** wrote a guide for the general public on the subject of advance directives concerning end-of-life, showing the interest of that instance for the subject.

B. Expected results and perspectives

Expected results are:

- to optimize care for people with mental diseases by placing them at the heart of their own care, to improve their awareness of their disorders, all the while controlling healthcare expenses, to specify the health and social efficiency of the "DaiP" program;
- to specify the efficiency of the "DaiP" program; the slight cost increase of the implemented program compared to the existing approach should be partially or even totally compensated by a better rationalization of healthcare expenses due to a reduced number of avoidable hospital admissions and the improvement of key social-health indicators for mental disorders: insight, empowerment, therapeutic alliance. The potential slight cost increase could also be justified by better recovery of patients and by a good cost-benefit ratio;
- to know and understand how resources are mobilized (PAD tool, peer-worker to facilitate its use), but also what consequences they have on follow-up, on the user's pathway, this being at the center of our preoccupations. The program's success certainly depends on the adherence of the users themselves, but also on its acceptability for the helpers, carers and the social body as a whole.
- To study limiting factors or those favoring the transposability of the strategy.

More than just answering the objectives of a research project, the project will be proposing an innovative approach base on recovery-oriented care constituting a true societal project where mental illness is no longer considered a disability but an experience during which the person can develop strengths and skills to fight against it, but also to accept and understand, control and use. Professionals must work with these skills and no longer only and mainly on difficulties. The aims is to valorize the person, his/her pathway, resilience, capacity and also his/her rights.

In the end, the objective of describing the program's effects on healthcare and social organizations and on the representations and practices of professionals and users will help to understand better under which conditions it would be possible to generalize this experimentation on a national scale.

C. Feasibility

The feasibility of the intervention and research is based on a preliminary study on six patients which resulted in a psychiatry thesis (71) and showed the importance for the users of having a facilitator-peer-worker present in the drafting of the document and of sharing it with the psychiatrist. It also led to the appropriation of the document by a users' organization, Eutopia, who perfected the document for a better acceptability of filling it in. Integrating the mastery of this document in the program of a recovery training center, Centre de Formation au Rétablissement (CoFoR-Marseille), since September 2017 helps homogeneous diffusion of this practice to peer-workers who will be in charge of assisting patients to fill out the document.
Finally, the proximity of the research actors with the field actors, through professional affiliation, previous and ongoing partnerships is a guarantee for the feasibility of the trial, in all its articulated sections.

D. Limitation and justification

1. Experimental plan

The experimental plan retained is based on a randomized experimental diagram. In the light

of discussions about the generalization of the approach it is important to propose the trial diagram that brings the highest level of formal proof and the most recognized.
This plan's limit is linked to the potential contamination of the control group. The knowledge of this potential bias could have justified a cluster study or a Zelen design (96). The fact that the patients were randomly drawn by the investigators avoided this bias but introduced a new major limit which is the extreme diversity of investigator practices concerning involuntary hospital admission (professional practices linked to the place of work or the specialization), thus the need to greatly increase the number of subjects in the trial. Zelen design is a methodology that has never been used in France, and which presents the particularity of only informing the person which experimental arm they are in. Using this technique, which would reduce access to advance directives for the control group, from an ethical point of view this presents the particularity of privileging the principle of benevolence (limiting the feeling of "missing a chance") over the principle of autonomy (being informed of the entire research project). Given the central importance of the notion of autonomy in this research, classical randomization was chosen.

2. Contagion of controls

The risk seems limited by the limited current knowledge about this approach. Publicity will be limited by the sample size in each city (50 people will fill in the document), the large number of psychiatrists involved (2 to 4 per site), ambulatory inclusion (versus hospital inclusion, where patients talk a great deal among themselves), and the narrow window of inclusion (6 months).

3. Absence of pairing

The implemented project does not offer pairing upon inclusion thus limiting comparability. The choice not to pair was based on the following observations:

- the factors of confusion to be taken into account have not yet been clearly defined;
- pairing according to variables such as age and gender would weigh down the procedure and limit inclusion;
- from an ethical point of view it seems difficult to propose pairing which requires prior randomization and which could lead to non inclusion in the project even though the population was solicited

The principle of prioritizing reality on the field, and the supposed or identified factors of confusion will be taken into account upon analysis as described in chapter VII. D. 3.

IX. Ethical and regulatory aspects

A. Risk-benefit ratio

1. Benefits and advantages

Benefits are on a community and an individual level. On a community level, this research will provide elements of reflection for public authorities concerned with caring for homeless people with mental disorders, in order to improve the health, the care pathway, the autonomy and the respect of the rights of this vulnerable population while managing to control expense.
On an individual level, patients included in the study benefit from a reimbursement of their

travel expenses resulting from their coming to the center for the visits, upon presentation of proof.
Since the interviews required for research are led by people trained to listen and support, the patient will be able to express him/herself on these occasions.
The beneficiaries of the experimental program will have access to an innovative approach, will be able to meet a peer-worker if they wish, which generally causes about a positive identification; they will be able to share information with their psychiatrist for less coercive future treatment.
The patients benefiting from usual care will benefit from regular meetings with the person leading research.

2. Risks and constraints

On an individual level, the risks for patients who are "beneficiaries of usual care" of participating in the trial are low. There can be a feeling of losing a chance when they are told they are in the control group, double-blinding being impossible. However there is no change in their usual care pathway, and the patients will not be prevented from drafting advance directives if they take their own initiative to do so. The constraints are related to the fact that they have to go to meetings scheduled with the interviewer.
The individual risks and constraints of the patients who are "beneficiaries of the experimental program" are related to the introspection necessary for the drafting, which can cause a painful evocation of the past, related to previous episodes of coercion; there is also the meeting with the peer-worker, who is an atypical professional, which could destabilize some patients depending on the representations and potential expectations that they may have concerning this meeting.
Another constraint is related to answering the survey questionnaires according to a calendar defined in advance with the interviewer.
On an individual level, a risk related to the use of psychiatric nosology at the time of inclusion for both groups of patients, the use of the stated illnesses can be painful for certain patients who are in denial of their disorders. This is a lesser risk in the experimental arm because of the presence of the peer-worker, who has an impact on destigmatization.

3. Risk-benefit ratio

Overall, the risk-benefit ratio corresponding to participating in this trial can be considered favorable.

B. Legal and ethical aspects

The **sponsor** for this project is represented by the **Assistance Publique des Hôpitaux de Marseille**. A regulatory watch will be carried out by the Sponsor. It will submit the project to the authorities for approval.

1. Institutional review board

This project is in the framework of **interventional research (category 2)** with minimal risks and constraints, according to terms in article L.1121-1 paragraph 2.

It is subject to a new regulatory measure which applies to research "involving human subjects" i.e. act n° 2012-300 of March 5, 2012 relating to research involving human subjects (called the Jardé act) as amended by Order n° 2016-800 of June 16, 2016, and its application decrees.

It is for this reason that it required the opinion of a **Comité de Protection des Personnes (Ethics committee).**

People under (reinforced) curatorship are purposely kept within the scope of the trial, for representativeness, and considering the importance of the benefit expected for these patients this justifies the predictable risk incurred, which is minimal, as are the constraints related to this research.
Indeed, the Code de Santé Publique (French public health regulations) states in article L. 1121-8 that adult persons under a measure of legal protection or incapable of expressing their consent may only be included for category 2 research if research of comparable efficiency cannot be carried out on another category of the population and with the following conditions: (1) either the importance of the expected benefit for these persons is such that it justifies the predictable risk incurred; (2) or this research is justified with regards to the expected benefit for other people in the same situation. In this case the predictable risks and the constraint of this research must be of minimal extent.

An **information sheet** will be handed to patients and **informed consent** will be collected from the person, information will also be given to the legal representative if the person is under curatorship. Information sheets and informed consent forms were drawn up in compliance with regulatory recommendations, stating namely the objective of the trial, the benefits and risks related to this trial, the trial procedure and all of the legal provisions to which the patients are entitled.

This research will be led according to good clinical practice, representing a group of quality requirements in the fields of ethics and science, which must be met during the planning, implementation, leading, monitoring, quality control, audit, data collection, analysis and expression of results. Respecting these good clinical practices guarantees the protection of rights, security and the protection of the people participating in this research and the preservation of their anonymity as well as the credibility and accuracy of the data and results of this research.

2. Data protection

Data collected during the trial will be computer processed according to the loi "Informatique et Libertés" (French data protection act) n°78-17 of January 6, 1978 (article 40), these data will only be transmitted to the sponsor and if appropriate to authorized health authorities, under conditions guaranteeing their confidentiality.

Concerning the computer processing of data relating to this project, which has the purpose of research in the field of health, it falls within the framework of legal requirements, particularly the act of August 9, 2004 and will only concern data that does not allow the direct or indirect identification of the people concerned. It will be carried out in compliance with reference methodology accredited by the Commission nationale de l'informatique et des libertés and established in concertation with the consultative committee for the processing of data in matters of research in the field of health, set up in order to simplify formalities (Deliberation n°2016-262 of July 21, 2016 on the modification of the reference methodology

for the processing of personal data operating in the framework of biomedical research. Reference methodology MR-001).

It will also be carried out in compliance with the General Data Protection Regulation (GDPR) n° 2016/679 in force on May 25, 2018. This data processing reposes on the execution of a mission of public interest for which the data controller is responsible (article 6.1.e of GDPR), in the field of public health for scientific research purposes (paragraphs i and j of article 9.2 of GDPR).

In compliance with legal requirements of the French data protection act (Act n°78-17 of January 6, 1978 amended by act n° 2004-801 of August 6, 2004), and article 13 of the GDPR n°2016/679 in force on May 25, 2018, the patient has a right to access, rectify and erase collected personal data as well as a right to limit and to object to the processing of their data. He/she also has the right to object to the transmission of this data, under the obligation of professional secrecy, susceptible of being used in the frame of this research and of being processed.

Medical and non-medical staff involved in this research are sworn to medical and professional secrecy with regards to patient data collected during the trial. Collected patient data remains strictly confidential. It will be kept in paper form in a locked premises. It will be entered on a computer and benefit from automatic processing. This computer processing will not permit direct or indirect identification of subjects. All of the data may only be consulted by the principal investigator and the Sponsor’s representatives, or be transmitted to the Approved Health Authorities if necessary. Subjects can access their computer file by applying to a physician at the center.

3. Stopping the trial

In the eventuality of finances being stopped due to an insufficient number of participants, the trial could be suspended by the Sponsor. In this case, follow-up visits scheduled with the interviewer could be canceled. However, advance directives that have been drafted and shared would not be canceled. They are a tool for crisis prevention for the person, his/her family as well as for his/her psychiatrist, and may be used in the same manner outside the frame of the trial.

4. Quality control and insurance

Quality insurance and control, under the Sponsor’s responsibility, will be carried out according to Good Clinical Practices in order to guarantee the respect of the integrity of collected data, patient protection, respect of the protocol and of the legislation in force during the entire period of patients’ inclusion and follow-up by a clinical research associate commissioned by the Sponsor.
The type and frequency of monitoring will be established according to the level of monitoring defined according to patient risk and will depend on the number of patients included, the frequency of inclusions and difficulties observed during trial operation (procedures validated by the quality work group from the FHF promotion which determines the level of monitoring to be carried out according to the risk for the subject – OECD Recommendation on the Governance of Clinical Trials, December 2012).

During this trial, the level of monitoring is classified as "minimal" with type A patient risk. Consent forms and the presence of a statement of participation in the source file are required. If one/several consent forms do not comply, files will be randomly monitored.

5. Data archiving

The investigator must keep all of the documents concerning the trial (source documents, signed informed consent forms,…) for a period of 15 years from the date the final report for the trial was signed.

C. Clinical trials vigilance

This research belongs to research involving human subjects with minimum risks and constraints, and in compliance with legislation in force, it is the investigator's liability to report any serious adverse event occurring during investigations according to the internal procedure for reporting a serious adverse event associated with healthcare in his/her institution.
The investigator must also specify that the patient is included in a clinical trial and accurately identify the name of the trial.
Indeed, for this category of research there is no longer any specific vigilance of the trial for the sponsor (art L1123-10 CSP, last paragraph).

X. Report and publications

At the end of the trial a report will be written. It will give details of all of the decisions inherent to the leading of the project and the results obtained.
This report will constitute the basis for related communications and publications that will be co-signed by the members of the assessment Steering Committee who will reference the origins of any aid received.

XI. Valorization and societal sensitization

This objective is that of spreading or transferring knowledge to various types of public: administrators and professionals working with people who receive mental health care, jurists, social workers, public and community organizations defending human rights. This will be translated by the organization of sensitization events.

XII. References

1. Thomas W, Znaniecki F. The Polish Peasant in Europe and America: A CLASSIC WORK IN IMMIGRATION HISTORY. Student edition. Urbana: University of Illinois Press; 1996. 152 p.

2. Bertaux D. Du récit de vie dans l'approche de l'autre, The life story as path towards the other. L'Autre. 2000;me 1(2):239-57.

3. Grard, J. « Approche(s) narrative(s) et récit à la première personne. Généalogie et politiques de l'enquête. ». 2017;

4. Paliroda, Krieg, Murdock, Havelock. A pratical guide to photovoice. 2009.

5. Boal A. Jeux pour acteurs et non-acteurs : pratique du théâtre de l’opprimé. La Découverte, Paris; 2004.

6. The Belmont Report [Internet]. HHS.gov. 1979 [cité 12 sept 2017]. Disponible sur: https://www.hhs.gov/ohrp/regulations-and-policy/belmont-report/index.html

7. Senon J-L, Jonas C, Voyer M. Les soins sous contrainte des malades mentaux depuis la loi du 5 juillet 2011 « relative au droit et à la protection des personnes faisant l’objet de soins psychiatriques et aux modalités de leur prise en charge ». Annales Médico-psychologiques, revue psychiatrique. 1 avr 2012;170(3):211-5.

8. Gridel J. L’acte éminemment personnel et la volonté propre du majeur en tutelle [Internet]. 2000 [cité 9 sept 2017]. Disponible sur: https://www.courdecassation.fr/publications_26/rapport_annuel_36/rapport_2000_98/deuxieme_partie_tudes_documents_100/tudes_theme_protection_personne_102/personnel_volonte_5853.html

9. Martha C. Nussbaum. The Fragility of Goodness: Luck and Ethics in Greek Tragedy and Philosophy - [Internet]. [cité 9 sept 2017]. Disponible sur: https://www.amazon.fr/Fragility-Goodness-Ethics-Tragedy-Philosophy/dp/0521794722

10. Amartya S. L’Idee De Justice. Paris: Editions Flammarion; 2012.

11. Appelbaum PS, Roth LH. Competency to consent to research: a psychiatric overview. Arch Gen Psychiatry. août 1982;39(8):951-8.

12. Sturman ED. The capacity to consent to treatment and research: a review of standardized assessment tools. Clin Psychol Rev. nov 2005;25(7):954-74.

13. In Azimi V, Hennion-Jacquet P, Koubi G. La régulation des pratiques contraignantes de soin en santé mentale : perspectives pour une approche interdisciplinaire. 229-245 p. (L’institution psychiatrique au prisme du droit. La folie entre administration et justice).

14. Coldefy M, Fernandez R. « Les soins sans consentement en psychiatrie : bilan après quatre années de mise en œuvre de la loi du 5 juillet 2011 ». Irdes, Questions d’économie de la santé. 2017;22.

15. Vidon G, Hardy-Baylé M-C, Younès N. Quelle place pour les soins sans consentement en ambulatoire ? À propos de l’enquête IDF sur les programmes de soins, What is the role of outpatient care without patient consent? Comments regarding the IDF enquiry on the hospital care programme, Resumen. L’information psychiatrique. 15 sept 2015;me 91(7):602-7.

16. Steinert T, Lepping P, Bernhardsgrütter R, Conca A, Hatling T, Janssen W, et al. Incidence of seclusion and restraint in psychiatric hospitals: a literature review and survey of international trends. Soc Psychiatry Psychiatr Epidemiol. sept 2010;45(9):889-97.

17. Salize HJ, Dressing H. Epidemiology of involuntary placement of mentally ill people across the European Union. Br J Psychiatry. févr 2004;184:163-8.

18. Husum TL, Bjørngaard JH, Finset A, Ruud T. A cross-sectional prospective study of seclusion, restraint and involuntary medication in acute psychiatric wards: patient, staff and ward characteristics. BMC Health Serv Res. 6 avr 2010;10:89.

19. Kisely SR, Campbell LA, Preston NJ. Compulsory community and involuntary outpatient treatment for people with severe mental disorders. Cochrane Database Syst Rev. 16 févr 2011;(2):CD004408.

20. Nyttingnes O, Ruud T, Rugkåsa J. 'It's unbelievably humiliating'—Patients' expressions of negative effects of coercion in mental health care. International Journal of Law and Psychiatry. 1 nov 2016;49:147-53.

21. Sibitz I, Scheutz A, Lakeman R, Schrank B, Schaffer M, Amering M. « Impact of coercive measures on life stories: qualitative study ». The British Journal of Psychiatry [Internet]. 2011; Disponible sur: http://bjp.rcpsych.org/content/bjprcpsych/199/3/239.full.pdf

22. Swanson JW, Swartz MS, Elbogen EB, Wagner HR, Burns BJ. Effects of involuntary outpatient commitment on subjective quality of life in persons with severe mental illness. Behav Sci Law. 2003;21(4):473-91.

23. Fistein E, Clare I, Redley M. Tensions between policy and practice: A qualitative analysis of decisions regarding compulsory admission to psychiatric hospital - ScienceDirect [Internet]. [cité 8 sept 2017]. Disponible sur: http://www.sciencedirect.com/science/article/pii/S0160252716300425

24. Wynn DR. Coercion in psychiatric care: clinical, legal, and ethical controversies. International Journal of Psychiatry in Clinical Practice. 1 janv 2006;10(4):247-51.

25. Bloch S, Green SA. An ethical framework for psychiatry. Br J Psychiatry. janv 2006;188:7-12.

26. Dourlens C. L'action publique à l'épreuve de la fragilité normative. HDR, Université Jean Monnet – Saint-Etienne [Internet]. 2010; Disponible sur: https://tel.archives-ouvertes.fr/tel-00570175/document

27. Chi MH, Hsiao CY, Chen KC, Lee L-T, Tsai HC, Hui Lee I, et al. The readmission rate and medical cost of patients with schizophrenia after first hospitalization - A 10-year follow-up population-based study. Schizophr Res. janv 2016;170(1):184-90.

28. Burns T, Rugkåsa J, Molodynski A, Dawson J, Yeeles K, Vazquez-Montes M, et al. Community treatment orders for patients with psychosis (OCTET): a randomised controlled trial. Lancet. 11 mai 2013;381(9878):1627-33.

29. Bleuler M. The long-term course of the schizophrenic psychoses. Psychological Medicine [Internet]. 1974; Disponible sur: http://ulf.ing-steen.se/BRETTSTAMS_DEBATTSIDOR/Psykiatrin_ar_inte_mogen_for_tvangsbehandling_i_oppenvarden_files/Long%20term%20course%20of%20schizophrenia.%20Bleulers%20study.pdf

30. Van Dorn RA, Scheyett A, Swanson JW, Swartz MS. Psychiatric Advance Directives and Social Workers: An Integrative Review. Soc Work. avr 2010;55(2):157-67.

31. Netgen. Comment appliquer les nouvelles «règles du jeu» médecin-patient ? L'exemple des directives anticipées et du représentant thérapeutique [Internet]. Revue Médicale Suisse. [cité 8 sept 2017]. Disponible sur: https://www.revmed.ch/RMS/2004/RMS-2500/24066

32. Bortolato B, Miskowiak KW, Köhler CA, Vieta E, Carvalho AF. Cognitive dysfunction in bipolar disorder and schizophrenia: a systematic review of meta-analyses. Neuropsychiatr Dis Treat. 17 déc 2015;11:3111-25.

33. Bellack AS. Scientific and Consumer Models of Recovery in Schizophrenia: Concordance, Contrasts, and Implications. Schizophr Bull. 1 juill 2006;32(3):432-42.

34. Strauss GP, Harrow M, Grossman LS, Rosen C. Periods of recovery in deficit syndrome schizophrenia: a 20-year multi-follow-up longitudinal study. Schizophr Bull. juill 2010;36(4):788-99.

35. Farkas M. The vision of recovery today: what it is and what it means for services. World Psychiatry. juin 2007;6(2):68-74.

36. Lysaker PH, Ringer J, Maxwell C, Mcguire A, Lecomte T. Personal narratives and recovery from schizophrenia. Schizophrenia Research. 2010;121:Pages 1-282.

37. Franck N., Guelfi J, Rouillon F. Schizophrénie et autres troubles psychotiques. Introduction. Paris; 2012. 255-266 p. (Manuel de psychiatrie, 2ème édition).

38. Hopper K. Rethinking Social Recovery in Schizophrenia: What A Capabilities Approach Might Offer. Soc Sci Med. sept 2007;65(5):868-79.

39. Greacen T, Jouët E, Collectif. Pour des usagers de la psychiatrie acteurs de leur propre vie : Rétablissement, inclusion sociale, empowerment. Toulouse: Erès; 2012. 344 p.

40. Tobias CR, Rajabiun S, Franks J, Goldenkranz SB, Fine DN, Loscher-Hudson BS, et al. Peer Knowledge and Roles in Supporting Access to Care and Treatment. J Community Health. déc 2010;35(6):609-17.

41. Austin E, Ramakrishnan A, Hopper K. Embodying Recovery: A Qualitative Study of Peer Work in a Consumer-Run Service Setting. Community Ment Health J. nov 2014;50(8):879-85.

42. Dhanda A. Legal Capacity in the Disability Rights Convention: Stranglehold of the Past or Lodestar for the Future? | Supported Decision Making. Syracuse Journal International Law and Commerce. 2007;34:429-62.

43. Eyraud B, Velpry L. La liberté d'aller et venir comme révélateur du tournant juridique des régulations du soin en santé mentale : une mise en prerspective « internationale ». RDSS; 2016.

44. Henderson C, Flood C, Leese M, Thornicroft G, Sutherby K, Szmukler G. Views of service users and providers on joint crisis plans: single blind randomized controlled trial. Soc Psychiatry Psychiatr Epidemiol. mai 2009;44(5):369-76.

45. Décret n°2006-119 du 6 février 2006 relatif aux directives anticipées prévues par la loi n° 2005-370 du 22 avril 2005 relative aux droits des malades et à la fin de vie et modifiant le code de la santé publique (dispositions réglementaires). 2006-119 févr 6, 2006.

46. IGAS directives anticipées, rapport 2015 [Internet]. Disponible sur: http://www.igas.gouv.fr/IMG/pdf/2015-111R_Dir_anticipees.pdf

47. Campbell LA, Kisely SR. Advance treatment directives for people with severe mental illness. Cochrane Database Syst Rev. 21 janv 2009;(1):CD005963.

48. Maître E, Debien C, Nicaise P, Wyngaerden F, Galudec ML, Genest P, et al. Les Directives Anticipées incitatives en Psychiatrie : revue de la littérature qualitative, état des lieux et perspectives. /data/revues/00137006/v39i4/S0013700613000067/ [Internet]. 14 sept 2013 [cité 8 sept 2017]; Disponible sur: http://www.em-consulte.com/en/article/835062

49. Gay C, Motte P. Un plan d'action en cas de crise [Internet]. 2012. p. 62-65. Disponible sur: http://ifsante.centredoc.fr/opac/index.php?lvl=notice_display&id=74381

50. Khazaal Y, Richard C, Matthieu-Darekar S, Quement B, Kramer U, Preisig M. Advance directives in bipolar disorder, a cognitive behavioural conceptualization. Int J Law Psychiatry. févr 2008;31(1):1-8.

51. Drozdek D. Les Directives Anticipées incitatives en Psychiatrie - RERO DOC [Internet]. [cité 8 sept 2017]. Disponible sur: https://doc.rero.ch/record/9524

52. Henderson C, Swanson JW, Szmukler G, Thornicroft G, Zinkler M. A Typology of Advance Statements in Mental Health Care. PS. 1 janv 2008;59(1):63-71.

53. Swanson J, Swartz M, Ferron J, Elbogen E, Van Dorn R. Psychiatric advance directives among public mental health consumers in five U.S. cities: prevalence, demand, and correlates. J Am Acad Psychiatry Law. 2006;34(1):43-57.

54. Swanson JW, Swartz MS, Elbogen EB, Van Dorn RA, Ferron J, Wagner HR, et al. Facilitated psychiatric advance directives: a randomized trial of an intervention to foster advance treatment planning among persons with severe mental illness. Am J Psychiatry. nov 2006;163(11):1943-51.

55. Sutherby K, Szmukler GI, Halpern A, Alexander M, Thornicroft G, Johnson C, et al. A study of « crisis cards » in a community psychiatric service. Acta Psychiatr Scand. juill 1999;100(1):56-61.

56. Homère, Notor, Lisle L de. L'Odyssée. Collection Classiques Abrégés. Paris: ECOLE DES LOISIRS; 1987. 165 p.

57. Hysick Y. « Ulysses clause » allows agents to direct treatment during psychiatric emergency [Internet]. tribunedigital-dailypress. [cité 8 sept 2017]. Disponible sur: http://articles.dailypress.com/2013-06-10/health/dp-nws-mental-health-advance-0610-20130610_1_mental-health-plan-advance-directive-health-care

58. Copeland M. « Wellness Recovery Action Plan. », Dummerston, VT: Peach Press; 1997. [Internet]. MentalHealthRecovery. 2015 [cité 8 sept 2017]. Disponible sur: http://mentalhealthrecovery.com/info-center/mental-illness-self-management-through-wellness-recovery-action-planning/

59. Henderson C, Flood C, Leese M, Thornicroft G, Sutherby K, Szmukler G. Effect of joint crisis plans on use of compulsory treatment in psychiatry: single blind randomised controlled trial. BMJ. 17 juill 2004;329(7458):136.

60. SWANSON JW, SWARTZ MS, ELBOGEN EB, VAN DORN RA, WAGNER HR, MOSER LA, et al. Psychiatric advance directives and reduction of coercive crisis interventions. J Ment Health. 1 janv 2008;17(3):255-67.

61. Papageorgiou A, King M, Janmohamed A, Davidson O, Dawson J. Advance directives for patients compulsorily admitted to hospital with serious mental illness. Randomised controlled trial. Br J Psychiatry. déc 2002;181:513-9.

62. Atkinson JM, Garner HC, Gilmour WH. Models of advance directives in mental health care: stakeholder views. Soc Psychiatry Psychiatr Epidemiol. août 2004;39(8):673-80.

63. Williams GC, Rodin GC, Ryan RM, Grolnick WS, Deci EL. Autonomous regulation and long-term medication adherence in adult outpatients. Health Psychol. mai 1998;17(3):269-76.

64. Nicaise P, Lorant V, Dubois V. Psychiatric Advance Directives as a complex and multistage intervention: a realist systematic review. Health Soc Care Community. janv 2013;21(1):1-14.

65. Gross R. Les enjeux des Directives Anticipées incitatives en Psychiatrie. févr 2011;

66. Advance directives based on cognitive therapy: A way to overcome coercion related problems (PDF Download Available) [Internet]. ResearchGate. [cité 8 sept 2017]. Disponible sur: https://www.researchgate.net/publication/23293874_Advance_directives_based_on_cognitive_therapy_A_way_to_overcome_coercion_related_problems

67. La Fond JQ, Srebnik D. The impact of mental health advance directives on patient perceptions of coercion in civil commitment and treatment decisions. Int J Law Psychiatry. déc 2002;25(6):537-55.

68. Srebnik DS, Russo J. Consistency of psychiatric crisis care with advance directive instructions. Psychiatr Serv. sept 2007;58(9):1157-63.

69. Widdershoven G, Berghmans R. Advance directives in psychiatric care: a narrative approach. J Med Ethics. avr 2001;27(2):92-7.

70. Maurel M. [Education and recurrence prevention]. Encephale. janv 2009;35 Suppl 1:S20-23.

71. Pontier M. Expérimentation d'un protocole de Directives Anticipées incitatives en Psychiatrie. [Thèse d'exercice : Médecine. DES de psychiatrie]. Aix-Marseille; 2016.

72. Janofsky JS, McCarthy RJ, Folstein MF. The Hopkins Competency Assessment Test: a brief method for evaluating patients' capacity to give informed consent. Hosp Community Psychiatry. févr 1992;43(2):132-6.

73. Barton C, Mallik H., Orr J, Janofsky J. Clinicians' judgement of capacity of nursing home patients to give informed consent. Psychiatric Services. 1996;956-60.

74. Guy W. ECDEU assessment manual for psychopharmacology. Rev. 1976. Rockville, Md: U.S. Dept. of Health, Education, and Welfare, Public Health Service, Alcohol, Drug Abuse, and Mental Health Administration, National Institute of Mental Health, Psychopharmacology Research Branch, Division of Extramural Research Programs; 1976. 603 p.

75. San L, Serrano M, Cañas F, Romero SL, Sánchez-Cabezudo Á, Villar M. Towards a pragmatic and operational definition of relapse in schizophrenia: A Delphi consensus approach. Int J Psychiatry Clin Pract. juin 2015;19(2):90-8.

76. Corrigan PW, Giffort D, Rashid F, Leary M, Okeke I. Recovery as a psychological construct. Community Ment Health J. juin 1999;35(3):231-9.

77. Corrigan PW, Salzer M, Ralph RO, Sangster Y, Keck L. Examining the factor structure of the recovery assessment scale. Schizophr Bull. 2004;30(4):1035-41.

78. Misdrahi D, Verdoux H, Lançon C, Bayle F. The 4-Point ordinal Alliance Self-report: a self-report questionnaire for assessing therapeutic relationships in routine mental health. Compr Psychiatry. avr 2009;50(2):181-5.

79. Amador XF, Flaum M, Andreasen NC, Strauss DH, Yale SA, Clark SC, et al. Awareness of illness in schizophrenia and schizoaffective and mood disorders. Arch Gen Psychiatry. oct 1994;51(10):826-36.

80. Michel P, Baumstarck K, Auquier P, Amador X, Dumas R, Fernandez J, et al. Psychometric properties of the abbreviated version of the Scale to Assess Unawareness in Mental Disorder in schizophrenia. BMC Psychiatry. 22 sept 2013;13:229.

81. Rogers ES, Chamberlin J, Ellison ML, Crean T. A consumer-constructed scale to measure empowerment among users of mental health services. Psychiatr Serv. août 1997;48(8):1042-7.

82. Auquier P, Simeoni MC, Sapin C, Reine G, Aghababian V, Cramer J, et al. Development and validation of a patient-based health-related quality of life questionnaire in schizophrenia: the S-QoL. Schizophr Res. 1 sept 2003;63(1-2):137-49.

83. Girard V, Tinland A, Boucekine M, Loubière S, Lancon C, Boyer L, et al. Validity of a common quality of life measurement in homeless individuals with bipolar disorder and schizophrenia. J Affect Disord. 1 nov 2016;204:131-7.

84. Attkisson CC, Zwick R. The client satisfaction questionnaire. Psychometric properties and correlations with service utilization and psychotherapy outcome. Eval Program Plann. 1982;5(3):233-7.

85. Conseil d'éthique clinique. « Application des Directives Anticipées incitatives en Psychiatrie : refus de traitements psychotropes en cas de décompensation psychiatrique »,. HUG (Hôpitaux Universitaires de Genève) [Internet]. Disponible sur: http://www.hug-ge.ch/sites/interhug/files/documents/soigner/ethique/directives-anticipees_psy.pdf

86. Brooks R. EuroQol: the current state of play. Health Policy. juill 1996;37(1):53-72.

87. Doise W. Droit de l'homme et force des idées. PUF; 2001.

88. Ewick P, Silbey S. The common place of law : stories of everyday life. Chicago University Press; 1998.

89. Le Marcis F, Grard, J. Everyday Ethics in a South African Medical Ward", in de Herdt, Tom; Olivier de Sardan, Jean-Pierre (Dir.). Real governance and practical norms in Sub-Saharan Africa: the game of the rules, Routledge. 2015;pp.160-185.

90. Fassin D. "The elementary forms of care An empirical approach to ethics in a South African Hospital". . Social Science & Medicine. 2008;67(2):262-70.

91. Eyraud B, Despres C. Usages et significations du droit des (in)capacités. Retraite et société; 2014.

92. Weber M, Chavy J. L'éthique protestante et l'esprit du capitalisme. Paris: Pocket; 1904. 285 p.

93. Wang C, Burris MA. Photovoice: Concept, Methodology, and Use for Participatory Needs Assessment. Health Educ Behav. 1 juin 1997;24(3):369-87.

94. OMS | Législation touchant la santé mentale et les droits de l'homme [Internet]. WHO. 2005 [cité 8 sept 2017]. Disponible sur: http://www.who.int/mental_health/policy/services/essentialpackage1v5/fr/

95. OMS. Guidelines for the promotion of human rights of persons with mental disorders. Geneva: World Health Organization. World Health Organization; 1996.

96. Zelen M. A New Design for Randomized Clinical Trials [Internet]. http://dx.doi.org/10.1056/NEJM197905313002203. 1979 [cité 9 sept 2017]. Disponible sur: http://www.nejm.org/doi/full/10.1056/NEJM197905313002203

97 Belanger A. Validation of a French-language version of the health education impact Questionnaire (heiQ) among chronic disease patients seen in primary care. 2015.

XIII. Annexes

A. Annex 1: French legal modes of care without consent in psychiatry since the act of July 5, 2011, amended September 27, 2013

According to the law of July 2011, 3 criteria are required for care without consent: the presence of mental health disorders, the impossibility to consent to care, the need of care and regular and constant medical supervision.

Psychiatric care by decision of the State's representative (SDRE) replaces and extend Involuntary Hospitalizations (HO) to other modalities like part-time and ambulatory care. A fourth criterion is required: the threat to personal safety or public order. The admission is pronounced by Prefectoral decree following a detailed medical certificate by a psychiatrist practising outside the hospital.

Psychiatric care at the request of a third party (SDT), formerly Hospitalisation at the request of a third party (HDT), is based on the notion of a "third party" allowing the patient to enter into care. Any person likely to act in the patient's interest who can prove a relationship prior to the admission (apart from the nursing staff) can be a "third party". The handwritten request for care must be completed by two detailed and concordant medical certificates. The first certificate must be issued by a doctor from outside the institution, while the second must be issued by a psychiatrist from the admitting hospital

Psychiatric care in case of imminent danger (SPI) was introduced by the law of July 5, 2011 in order to facilitate care for isolated or marginalized persons. The admission criteria are similar to those for people admitted in SDT. In addition, the absence of a third party who can be mobilized in the event of serious and imminent danger to the health and/or life of the person is an essential prerequisite for his or her admission, based on a decision by the director of the hospital, motivated by a medical certificate drawn up by a doctor who does not practice in the reception structure. The director has 24 hours to inform the patient's family or any person likely to act in his or her interest.

The following two types of care benefit from the same regime as admissions ordered by the State representative: **psychiatric care for persons judged to be criminally irresponsible (PJPI)** is part of a specific system with reinforced monitoring.

Psychiatric care for prisoners (D398): this legal mode of care was introduced before the reform of 2013 was implemented. It is specific to prisoners with severe mental disorders who require hospitalization and who cannot stay in prison. Using D398, the care is provided within a traditional psychiatric department. Since 2010, specially adapted hospital units (UHSA) allows prisoners to be hospitalized "freely" under specifically adapted conditions when these units exist in the area.

Temporary placement orders (OPP) concern minors when their parents are opposed to essential therapeutic care. The doctor can notify to the public prosecutor, who then submits the case to the Juge des Enfants (juvenile court judge) for a decision.

B. Annex 2: International political and legal context

- **United Nations**

1. **Convention on the Rights of Persons with Disabilities**

The United Nations Convention on the Rights of Persons with Disabilities is an international convention to promote, protect and fulfil the dignity, equal right, human rights and fundamental freedoms of people with all kinds of disabilities.
The Convention aims to change attitudes and approaches to persons with disabilities. It takes to a new height the movement from viewing persons with disabilities as "objects" of charity, medical treatment and social protection towards viewing persons with disabilities as "subjects" with rights, who are capable of claiming those rights and making decisions for their lives based on their free and informed consent as well as being active members of society. The Convention was as adopted on 2006 at the United Nations Headquarters and entered into force on 2008. France has ratified this convention, like more than 160 countries.

Article 12

Equal regognition before the law

1. States Parties reaffirm that persons with disabilities have the right to recognition everywhere as persons before the law.

2. States Parties shall recognize that persons with disabilities enjoy legal capacity on an equal basis with others in all aspects of life.

3. States Parties shall take appropriate measures to provide access by persons with disabilities to the support they may require in exercising their legal capacity.

4. States Parties shall ensure that all measures that relate to the exercise of legal capacity provide for appropriate and effective safeguards to prevent abuse in accordance with international human rights law. Such safeguards shall ensure that measures relating to the exercise of legal capacity respect the rights, will and preferences of the person, are free of conflict of interest and undue influence, are proportional and tailored to the person's circumstances, apply for the shortest time possible and are subject to regular review by a competent, independent and impartial authority or judicial body. The safeguards shall be proportional to the degree to which such measures affect the person's rights and interests.

5. Subject to the provisions of this article, States Parties shall take all appropriate and effective measures to ensure the equal right of persons with disabilities to own or inherit property, to control their own financial affairs and to have equal access to bank loans, mortgages and other forms of financial credit, and shall ensure that persons with disabilities are not arbitrarily deprived of their property.

2. **Convention against torture and Other Cruel, Inhuman or Degrading Treatment or Punishment CAT/C/FRA/CO/7**

In June 2016, le Committee considered the seventh periodic report of France. Psychiatric facilities were among the principal subjects of concern of the Committee, which stated (p6):

«

Psychiatric facilities

29. The Committee is concerned about: (a) the substandard physical conditions of detention of patients in certain psychiatric facilities; (b) the frequent use of seclusion for days on end, whether with or without restraint, notwithstanding the Act of 26 January 2016 on the modernization of the health system and the good practice recommendations issued by the French National Health Authority in 1998 and 2004; (c) the lack of a register on the use of seclusion and restraint, as provided for by the Act of 26 January 2016; (d) the fact that mechanical restraint is not always used according to the same criteria or for the same duration; and (e) the failure to systematically inform patients about their rights prior to placement in seclusion or under restraint and about how to appeal such decisions. While noting the State party's explanations of the measures taken, the Committee is particularly concerned about the findings of the Inspector General of Places of Deprivation of Liberty on the use of seclusion and restraint in the Ain Psychotherapy Centre and the physical conditions in which the persons committed there are kept (arts. 11 and 16).

30. The Committee recommends that the State party:
(a) Improve the conditions of detention of persons committed to psychiatric hospitals;
(b) Ensure that there is no systematic or excessively frequent use of seclusion and restraint;
(c) Enhance the training of personnel at psychiatric facilities to ensure effective compliance with the rules established by the Act of 26 January 2016 on the modernization of the health system and the good practice recommendations issued by the National Health Authority in 1998 and 2004;
(d) Ensure that, in all cases of involuntary hospitalization, the Act of 5 July 2011, as amended on 25 September 2013, is properly enforced in connection with the oversight of such hospitalization and that patients are informed of their rights and of the avenues available to appeal such a decision.
»

C. Annex 3: Act of February 2016 on advance directives {Article L.1111-11 of the Code de la Santé Publique (CSP; French public health regulations)} and healthcare agents {Article L.1111-6 of CSP}

Any adult in France can designate a health care agent: the trusted person. It is a right offered to you, but it is not an obligation (Article L.1111-6 of the Public Health Code).

What is his/her role ?
The trusted person can perform the following tasks:

- **If you can express your will, he/she can accompany you in your steps and assist you during your medical appointments**

He/She can, if you wish :
- help you in your decisions concerning your health ;
- accompany you in your steps related to care;
- attend consultations : he/she assists you but does not replace you.
- take note of elements of your medical records in your presence : he/she will not have access to the information outside your presence.

- **In the event that your medical condition no longer allows you to give your opinion or make your decisions, the doctor or the medical team will consult the trusted person.**

His/Her opinion guides the doctor to make his decisions. He/she must therefore know your wishes and express them when called upon to do so.
The trusted person may be consulted by the team that treats you in case you are unable to express your will regarding the care provided to you and shall receive the necessary information to do so. In these circumstances, no significant intervention may be carried out without prior consultation except in cases of emergency or inability to reach him/her.

He/she will be your spokesperson to accurately reflect your wishes and will. Her testimony will prevail over all others.
He/she will transmit your advance directives to healthcare professionals if necessary.
She will not be responsible for making decisions about your treatment but will bear witness to your wishes: the responsibility belongs to the doctor and the decision will be made after consultation with another doctor and the health care team.

Warning: the trusted person should not be confused with *person to prevent*, who is alerted by telephone if your health condition worsens. But person designated as trusted person may also be that designated as person to prevent if necessary.

Who can be the trusted person ?
Anyone close to you (parent, close relative, attending physician) you trust and who agrees to play this role can be designated trusted person. It doesn't have to be someone in your family.
If you are under guardianship, you can designate a trusted person with the permission of the judge or family council if it has been constituted. If the person of trust has been designated

before the guardianship measure is in place, the family council or the judge may confirm or cancel the appointment of that person.

When to designate him/her ?
You can designate your trusted person at any time.
It is important that you discuss your wishes with him/her so that he/she understands your choices and your wishes. She would not express her/him own convictions, but yours, and should make a moral commitment to you to do so.

How can I designate him/her ?
This designation is made in writing, upon admission or during hospitalisation, on the forms that will be given to you for this purpose. This designation may also be made on free paper.

The designation may be cancelled or amended at any time.

Your attending physician should ensure that you are aware of the possibility to designate a trusted person. It prompts you to designate a trusted person if you didn't.

D. Annex 4: Human resources for the interventional research on each site

- One peer-worker in charge of facilitation of DAiP. This peer-worker will be welcomed in a healthcare team that is already used to include peer-workers, in order to facilitate the sharing of experience and self-help. The peer-workers of the 3 sites will be trained to facilitate DAiP by peer-workers of Eutopia team in the CoFoR Recovery College. This training will include assistance in writing one’s own advance directives. Regular telephone and physical support will be organized by Eutopia.

- One research assistant will be in charge of the coordination of stakeholders at the site’s level. He/she will be in contact with all psychiatrists to inform them, to remind them the inclusion criteria and the study procedures such as randomisation or consent form. He/she will meet with the eligible participants and validate inclusion and exclusion criteria. He/she will encourage and facilitate the meeting with the peer-worker, and the sharing of PADs with entourage and health care professionals. With the sociologist, he will be able to be part of the participatory research (Photovoice).

- The PI will be in charge of the coordination between sites through weekly online meeting.

E. Annex 5: Document "Directives Anticipées incitatives en Psychiatrie" "Psychiatric advance directives" developed by the organization Eutopia – *(Document not recuperated by the coordinating center and for data analysis)*

Psychiatric advanced directives
- *Eutopia* model

Person concerned and health care agent

I, undersigned ,...,
born on............................... in ...., designates as health care agent in accordance with the decree L. 311-5-1 of the Code of Social Action and Families:

	Contact	Type of relationship	Function
M.-Mrs.			
M.-Mrs.			
Others			

To ensure the carrying out the of my following Psychiatric Advance Directives, with my consent at the time of writing.
It is understood that the Psychiatric Advance Directives are not binding on relatives and caregivers.

Done in .... the ....../......./......./........., alone or with ....

Name(s) : Signature(s) :

- When I am competent to decide:	- When I am not competent to decide:

Warning signs

1 –

3 –

2 –

4 –

New signs (« update ») :

____

____

____

What help me in case of crisis :

1 –

3 –

2 –

4 –

What doesn't help me in case of crisis :

1 –

3 –

2 –

4 –

Signals of end of crisis, when it gets better:

1 –

3 –

2 –

4 –

In case of danger (auto or hetero agressivity)

To do :
-
-
-

Not to do :
-
-
-

I want to take a medication: YES □ NO □

Medications that help me, that can be used

Medication	Dosage	Effect	Note

Medications that do not help me, that should not be used

Medication	Dosage	Effect	Note

I want to be accompanied to a care setting: YES □ NO □

Care settings I want to use (hospitals, services, teams)

-
-
-
-
-
-

Remarks :
....
....
....

Care settings I don't want to use :

-
-
-
-
-
-

Remarks:
....

....
....

Health care professionals

- I want to be supported by :

*
*
*

- I don't want to be supported by :

*
*
*

Other personal remarks, useful tips, notes…

....
....
....
....
....
....
....
....
....
....
....
....
....
....
....
....
....
....
....
....
....
....
....

F. Annex 6: Information sheet intended for professionals

Document d'information concernant la recherche
« Directives Anticipées indicatives en Psychiatrie (DAiP) »

Study rationale:
Some severe mental illnesses are characterized by symptomatic fluctuation accompanied by more or less severe cognitive alteration that can affect capacity. Some episodes can lead to **involontary hospital admissions**. Because of the health, social and economic impact of involuntary hospitalization, and because of the importance of self-determination and empowerment in the development of these disorders, new strategies have been developed to better consider the opinions of individuals. **Psychiatric Advance Directives** are one of these strategies. Advance directives are set up by a person who still has decision-making capacities, but who is anticipating potential incapacity, potential decline of their functional capacities. Their goal is to give the opinion of a person who is no longer capable of expressing themselves due to incapacity, to **represent the expression of a patient's free will**.

Studies have shown that PADs can develop the user's capacity to exercise a certain control over his/her care, involves him/her in a **process of appropriation** of his/her disorders, and in fine, modifies not only his/her course of care, but also his/her life course. In other words, it is a tool for "**advanced recovery education**" and **early prevention of relapses**, from a medical point of view; at the same time, it is **a means of (re)gaining some perspective on one's experience, a grip on one's existence**.

At international level, PADs are considered as **promising tools to reduce coercion and improve therapeutic alliance**, but **there is currently not enough data to formulate clinical recommendations**.

We formulate the hypothesis that the implementation of Psychiatric Advance Directives facilitated by peer-workers for people suffering from severe psychiatric disorders will reduce at short-term the rate of involuntary hospital admissions in the care pathway of these people, compared to individuals who have not benefited from this program.

This study is a **randomized controlled trial on 3 sites**: Lyon, Marseille et Paris. The quantitative component of the research will be completed by a socio-political qualitative component, and a participatory research component which aims at opening spaces for discussion between participants, researchers and professionals.

The people included in this research are followed for 1 year.
They meet with a research assistant every 6 months to answer a questionnaire.

Care strategies under study :
- For participants randomly allocated to the control group after randomization, the contents, frequency and process of consultations with the psychiatrist remain the same
- Participants randomly allocated to the Psychiatric advance directives facilitated by peer-worker group will be incited to fill a document called "Psychiatric Advance Directives"

and to meet a peer-worker to facilitate the filling. The document will contain the designation of the trusted person and care preferences of the participant in case of decompensation leading to abolition of judgment. The peer-worker will encourage the sharing of the document with the trusted person and with the psychiatrist or other healthcare professionals. The peer-worker will propose to accompany these moments of exchange.

Study population : Inclusion criteria are : aged over 18 ; presenting a diagnosis of schizophrenia, bipolar-I disorder or schizoaffective disorders according to DSM-IV classification ; benefiting from ambulatory follow-up by a referring psychiatrist, investigator ; having had involontary hospital admission at least once in the year prior to inclusion ; having decision-making capacity (HCAT score over 3) ; being capable of reading and writing ; étant capable de lire et écrire ; possibility of being under curatorship; affiliated to a social protection scheme ; accepting to participate in the trial and with informed consent signed by the individual.

Expected results and consequences: This research will allow to assess the effectiveness, efficiency and impact of the « Psychiatric Advance Directives » program in comparison with conventional psychiatric follow-up alone; to propose any necessary adaptation to the intervention model. Ultimately, the objective of describing the effects of the program on health organizations and on the representations and practices of professionals, caregivers, and user sis to provide a better understanding of the feasibility of generalizing this experiment.

Notice: Psychiatric advance directives incite to follow the preference and choices of the people in question, but the carers are not legally bound to provide the care requested if it does not correspond to practice standards.
PAD do not have any binding value for relatives and carers.

In France, advance directives are only binding in end-of-life healthcare.
The designation of the trusted person is framed by the article L. 311-5-1 of the French Public Health Regulation.

For any question about this project, you can contact :

- Research coordinator
  - o Senior sociologist in charge of qualitative research. Tel. Mail.
- Site of Marseille
  - o Investigator in charge of the site : Dr Magali Pontier - Tel. Mail.
  - o Research assistant : M. x.x. Tel : . Mail : .
  - o Peer-worker: M. x.x. Tel : . Mail.
- Site of Lyon
  - o Investigator in charge of the site : Dr Edouard Leaune - Tel. Mail.
  - o Research assistant: M. x.x.. Tel : . Mail : .
  - o Peer-worker: M. x.x. Tel : . Mail.
- Site of Paris
  - o Investigator in charge of the site : Dr Tim Greacen - Tel. Mail.
  - o Research assistant: M. x.x. Tel : . Mail : .

- o Peer-worker: M. x.x. Tel : . Mail.

Sponsor :

AP-HM
Assistance Publique des Hôpitaux de Marseille, 80 Rue Brochier, 13354 MARSEILLE Cedex 5

Principal investigator : Dr Aurélie TINLAND
Pôle psychiatrique universitaire Solaris, hôpital Sainte-Marguerite, APHM, 261 Bd Sainte Marguerite, 13009 Marseille.
EA 3279 : CEReSS - Centre d'Etude et de Recherche sur les Services de Santé et la Qualité de vie, Aix-Marseille Université, Faculté de Médecine - Secteur Timone, 27 bd Jean Moulin, 13005 Marseille
