# Supplementary material for: Effect of Psychiatric Advance Directives Facilitated by Peer Workers on Compulsory Admission Among People With Mental Illness: A Randomized Clinical Trial
Source: JAMA Psychiatry. 2022 Jun 6;79(8):752–9. doi: 10.1001/jamapsychiatry.2022.1627 (PMC9171654; doi:10.1001/jamapsychiatry.2022.1627)
Supplement: Supplement 3. — Collaborators in the DAiP group [file jamapsychiatry-e221627-s003.pdf]

Supplemental Online Content: Nonauthor Collaborators

*Indicates required information. Only first name, last name, and suffix will appear in PubMed.

*Group Name(s): DAiP group							
***First Name and Middle Initial(s)**	***Last Name**	***Suffix (eg, Jr, III)**	Academic Degrees	Institution	Location (city, state/province, country)	Role or Contribution, eg, chair, principal investigator	Group (if more than 1 Group listed in the byline) and/or Subgroup (eg, Steering Committee)
Lee	Antoine			Centre Hospitalier Le Vinatier	Lyon, France	Peer worker, member of the steering commitee	
Elsa	Castot			Assistance Publique Hopitaux de Marseille	Marseille, France	Peer worker, member of the steering commitee	
Nicolas	Grand Ordener			Assistance Publique Hopitaux de Marseille	Marseille, France	Coordination of the co-building stage, peer worker, member of the steering commitee	
Julien	Grard		PhD	Assistance Publique Hopitaux de Marseille	Marseille, France	Coordination of the co-building stage, member of the steering commitee	
Juliette	Robert			Assistance Publique Hopitaux de Marseille	Marseille, France	Research assistant, member of the steering commitee	
Antoine	Simon			Centre Hospitalier Le Vinatier	Lyon, France	Research assistant, member of the steering commitee	
Aurélien	Troisoeufs		PhD	SM-SHS, GHU-Paris Neurosciences	Paris, France	Member of the steering commitee	
