# Supplementary material for: Effect of Psychiatric Advance Directives Facilitated by Peer Workers on Compulsory Admission Among People With Mental Illness: A Randomized Clinical Trial
Source: JAMA Psychiatry. 2022 Jun 6;79(8):752–9. doi: 10.1001/jamapsychiatry.2022.1627 (PMC9171654; doi:10.1001/jamapsychiatry.2022.1627)
Supplement: Supplement 4. — Data sharing statement [file jamapsychiatry-e221627-s004.pdf]

Data Sharing Statement

Data
Data available: No
